# Immune-inflammatory profiles and disease severity in pulmonary tuberculosis complicated by central nervous system tuberculosis

**DOI:** 10.3389/fcimb.2026.1899254

**Published:** 2026-09-09

**Authors:** Muxing Chen, Di Wu, Xiaohong Chen, Jiandong Lin, Youfei Lin, Hongru Li

**Affiliations:** 1 Shengli Clinical Medical College of Fujian Medical University, Fujian Provincial Hospital, Fuzhou University Affiliated Provincial Hospital, Fuzhou, China; 2 Department of Tuberculosis, Fuzhou Pulmonary Hospital of Fujian, Fuzhou, China

**Keywords:** central nervous system tuberculosis, cerebrospinal fluid, immunoinflammation, neutrophil-to-lymphocyte ratio, pulmonary tuberculosis, T-lymphocyte subsets

## Abstract

**Objective:**

To compare peripheral blood immune–inflammatory profiles between patients with pulmonary tuberculosis complicated by central nervous system tuberculosis (PTB + CNS-TB) and those with pulmonary tuberculosis alone (PTB), and to identify laboratory parameters associated with disease severity in PTB + CNS-TB.

**Methods:**

This retrospective study included 4,829 inpatients with tuberculosis (January 2018–March 2026), comprising a PTB group (*n* = 4,578) and a PTB + CNS-TB group (*n* = 251). Propensity score matching (1:4) was performed using sex, age, body mass index, albumin, and hemoglobin, yielding 246 PTB + CNS-TB patients and 954 matched PTB controls. PTB + CNS-TB cases were further stratified by Medical Research Council (MRC) grading (Grade I: *n* = 165; Grades II–III: *n* = 86). Covariate-adjusted analyses with FDR correction were used to screen severity-associated parameters; LASSO and Firth’s logistic regression were applied to identify mutually independent factors and to construct a severity assessment model.

**Results:**

After matching, PTB + CNS-TB patients exhibited a distinct bidirectional pattern characterized by reduced peripheral lymphocyte counts and lower peripheral T-cell counts (CD3^+^, CD4^+^, and CD8^+^) and CD45^+^ lymphocytes counts, alongside increased neutrophil-related inflammatory indices (including neutrophil count, neutrophil-to-lymphocyte ratio, platelet-to-lymphocyte ratio, and systemic immune-inflammation index) (all P < 0.05). Within the PTB + CNS-TB cohort, severe disease was associated with higher white blood cell and neutrophil-related measures (notably NEU% and NLR) and more pronounced cerebrospinal fluid (CSF) abnormalities, particularly elevated CSF protein and decreased CSF chloride, whereas peripheral T-cell subset parameters did not differ significantly by severity. Multivariable modeling identified CSF protein, NEU%, and albumin as mutually independent factors associated with severe disease. A combined model including these three markers showed good internal discrimination (AUC = 0.772; bootstrap-corrected AUC = 0.767) and calibration.

**Conclusions:**

PTB + CNS-TB is associated with reduced peripheral lymphocyte/T-cell subset counts accompanied by heightened a neutrophil-predominant inflammatory profile. Among PTB + CNS-TB patients, disease severity shows a closer association with neutrophil-related indices and routine CSF parameters, particularly CSF protein, than with peripheral T-cell subset counts. A model incorporating NEU%, CSF protein, and albumin may aid in severity assessment.

## 1 Introduction

Tuberculosis (TB) remains one of the most formidable infectious threats to global public health. According to the latest data from the World Health Organization, an estimated 10.7 million new TB cases were recorded worldwide in 2024, with 1.23 million deaths, sustaining TB’s position as the leading cause of mortality from a single infectious agent (World Health Organization, 2025a). China ranks among the 30 high-burden countries, accounting for approximately 6.5% of global incident cases in 2024, a disease burden that demands continued vigilance (World Health Organization, 2025a). Among the diverse clinical manifestations of TB, central nervous system tuberculosis (CNS-TB) represents one of the most devastating forms of extrapulmonary disease (Abera et al., 2024; Plaatjie et al., 2025; Garcia et al., 2026). A systematic review and meta-analysis reported a case fatality rate of approximately 24.7% among adults with tuberculous meningitis, with roughly 50.9% of survivors sustaining neurological sequelae of varying severity (Wang et al., 2019). These figures underscore a sobering reality: even patients who survive CNS involvement frequently face the long-term burden of irreversible neurological disability.

Pathogenetically, *Mycobacterium tuberculosis* can disseminate hematogenously from a primary pulmonary focus, breach the blood-brain barrier, and invade the central nervous system, thereby triggering a profound intracranial inflammatory cascade (Davis et al., 2019). This process of neurotropic spread not only substantially complicates the clinical course but may also engender immunoinflammatory profiles that differ fundamentally from those observed in pulmonary tuberculosis (PTB) alone. Despite its clinical significance, the overall immunoinflammatory landscape of PTB complicated by CNS-TB (PTB + CNS-TB) as a distinct patient population, and in particular its systematic comparison with uncomplicated PTB, remains incompletely characterized.

In recent years, composite inflammatory indices derived from routine peripheral blood cell counts have attracted considerable attention in the assessment of infectious diseases. The neutrophil-to-lymphocyte ratio (NLR), platelet-to-lymphocyte ratio (PLR), lymphocyte-to-monocyte ratio (LMR), and systemic immune-inflammation index (SII) are widely regarded as integrated reflections of the dynamic equilibrium between pro- and anti-inflammatory responses during infection (Ninla-Aesong et al., 2024; Vitiello et al., 2024), with preliminary applications reported in the TB setting (Gegin et al., 2025; Chen et al., 2026). Equally central to the evaluation of cellular immune competence are T-lymphocyte subsets (CD3^+^, CD4^+^, and CD8^+^ T cells) and CD45^+^ lymphocytes counts. The Th1-polarized immune response mediated by CD4^+^ T cells plays a pivotal role in protective anti-TB immunity, while CD8^+^ T cells contribute to mycobacterial clearance through cytotoxic mechanisms and interferon-γ secretion (Lin and Flynn, 2015; Vats et al., 2024; Liu et al., 2025). How the host immunoinflammatory milieu is reorganized when *M. tuberculosis* extends its reach to the central nervous system remains a question for which large-scale evidence is lacking.

For the clinical assessment of disease severity, the Medical Research Council (MRC) grading system has long served as the standard tool for stratifying the severity of tuberculous meningitis. Based on Glasgow Coma Scale scores and the presence of focal neurological deficits, patients are classified into three grades (I, II, and III), with Grade III indicating critical illness (STREPTOMYCIN, 1948; Thwaites and Tran, 2005; Tuberculous Meningitis Committee of Chinese Tuberculosis Association, Chinese Medical Association, 2020). Prior studies have established a close association between MRC grade and patient outcomes (Thwaites et al., 2004); however, the independent predictors that distinguish mild from severe PTB + CNS-TB disease, particularly the combined prognostic value of peripheral blood immunoinflammatory markers and cerebrospinal fluid (CSF) parameters, have yet to be systematically examined.

Against this backdrop, the present study enrolled 4,829 hospitalized TB patients from Fuzhou Pulmonary Hospital, Fujian Province, between January 2018 and March 2026. Through propensity score matching (PSM) to control for intergroup confounding, we systematically compared peripheral blood immunoinflammatory profiles between PTB + CNS-TB patients and those with PTB alone. Using the MRC grading criteria as the reference standard, we further examined the covariate-adjusted associations between peripheral blood indices, CSF parameters, and CNS-TB severity, addressed collinearity among correlated inflammatory indices to identify independently associated parameters and constructed and internally validated a combined severity-assessment model.

## 2 Methods

### 2.1 Study population

**Figure 1:**
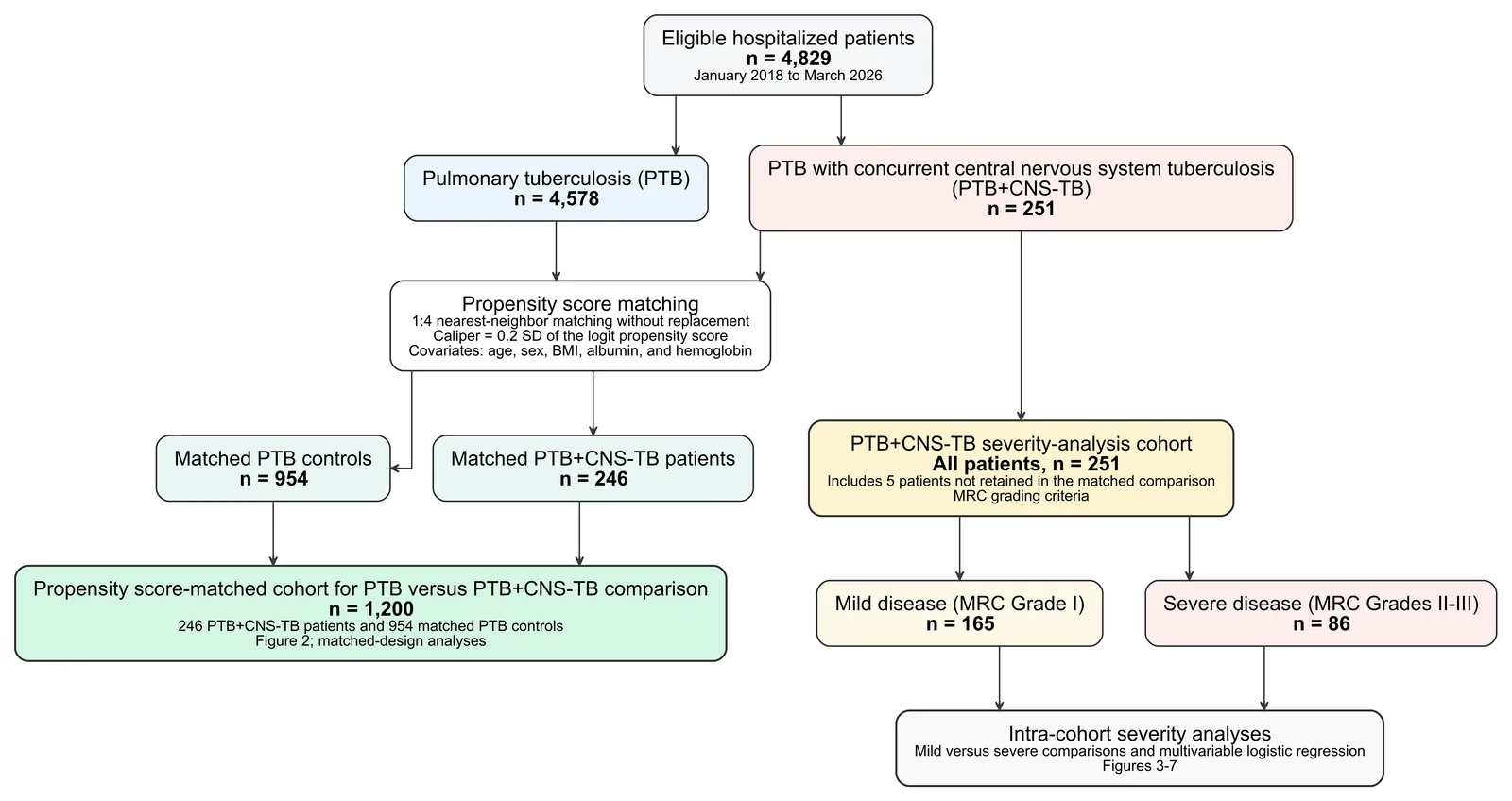
Study flow. The propensity score–matched comparison included 246 PTB+CNS-TB patients and 954 PTB controls. All 251 PTB+CNS-TB patients, including 5 patients not retained after matching, were included in the MRC-based severity analyses.

This single-center retrospective cohort study was conducted at Fuzhou Pulmonary Hospital of Fujian Province, China, enrolling patients admitted between January 1, 2018, and March 31, 2026. Following application of pre-specified eligibility criteria, a total of 4,829 patients were included in the final analytical dataset and stratified into two mutually exclusive cohorts: a pulmonary tuberculosis (PTB) cohort comprising 4,578 patients with isolated pulmonary disease, and a PTB with concurrent central nervous system tuberculosis (PTB + CNS-TB) cohort comprising 251 patients. Patients were eligible if they: (1) were aged ≥ 18 years; and (2) had a confirmed diagnosis of PTB, CNS-TB, or both. Exclusion criteria were: (1) recent receipt of interventions with potential immunomodulatory effects, including immunosuppressive agents or systemic corticosteroids; (2) known conditions affecting immune homeostasis, including human immunodeficiency virus (HIV) infection, autoimmune disorders, concurrent active infections other than tuberculosis, or malignancy; (3) incomplete clinical records (defined as missing data for complete blood count, serum biochemistry, T lymphocyte subsets, or CSF routine and biochemical parameters in the PTB + CNS-TB cohort); (4) pulmonary tuberculosis with any extrapulmonary involvement (applicable to the PTB cohort); or (5) absence of concomitant pulmonary tuberculosis (applicable to the PTB + CNS-TB cohort). Of note, to ensure cohort homogeneity and prioritize the evaluation of cerebrospinal fluid profiles, this study specifically focused on tuberculous meningitis; therefore, patients with isolated intracranial tuberculoma or isolated spinal tuberculosis lacking clinical or laboratory evidence of meningitis were also excluded. PTB was diagnosed in accordance with WHO guidelines (World Health Organization, 2025b) and the Chinese national tuberculosis prevention and control technical guidelines (National Center for Tuberculosis Prevention and Control, Chinese Center for Disease Control and Prevention, 2021), based on microbiological confirmation (*Mycobacterium tuberculosis* smear microscopy, culture, or nucleic acid amplification testing), characteristic chest computed tomographic findings, and/or documented response to standardized anti-tuberculosis therapy. CNS-TB was diagnosed on the basis of either (Tuberculous Meningitis Committee of Chinese Tuberculosis Association, Chinese Medical Association, 2020; National Center for Tuberculosis Prevention and Control, Chinese Center for Disease Control and Prevention, 2021): (i) microbiological evidence (positive *M. tuberculosis* culture or tuberculosis nucleic acid detection from CSF); or (ii) clinical-radiological criteria, comprising characteristic neuroimaging findings in conjunction with CSF abnormalities (elevated protein concentration, reduced glucose and chloride levels) and a positive response to anti-tuberculosis treatment. To further delineate the determinants of disease severity within the PTB + CNS-TB cohort, patients were stratified according to the MRC grading system, with Grade I classified as mild disease (*n* = 165) and Grades II–III collectively classified as severe disease (*n* = 86) (**Figure 1**). MRC severity grading was performed by senior clinicians based on standardized neurological assessment criteria applied at admission. All clinical data were retrospectively extracted from the institutional electronic medical records system by two independent trained investigators; discrepancies were adjudicated through consensus discussion.

### 2.2 Variables

Study data encompassed baseline demographic characteristics (age, sex), body mass index (BMI), and comprehensive laboratory parameters. All peripheral blood specimens were collected within 24 hours of admission and prior to the initiation of anti-tuberculosis therapy and corticosteroids, ensuring that measurements reflected the patient’s baseline immunological and inflammatory status unconfounded by treatment effects. Hematological indices, including white blood cell count (WBC), absolute neutrophil count (NEU), neutrophil percentage (NEU%), lymphocyte (LYMPH) count, monocyte count (MONO), red blood cell count (RBC), hemoglobin (Hb), and platelet count (PLT), were measured using a standardized automated hematology analyzer. Serum albumin (ALB) was determined by the bromocresol green colorimetric method. T lymphocyte subsets (CD3^+^, CD4^+^, and CD8^+^) and CD45^+^ lymphocytes were analyzed by flow cytometry from the first venous blood sample collected after admission. T-lymphocyte subsets and CD45^+^ lymphocytes were assessed using the BD MultiTEST IMK Kit, which included CD3-FITC (clone SK7, 2.3 μg/mL), CD8-PE (clone SK1, 1.75 μg/mL), CD45-PerCP (clone 2D1, 7.5 μg/mL), and CD4-APC (clone SK3, 0.92 μg/mL). A stepwise gating procedure was used for subset identification. Leukocytes were initially gated according to CD45 expression and side-scatter characteristics. Lymphocytes were then selected from the CD45^+^ leukocyte population using their forward- and side-scatter profiles. CD3^+^ cells were identified as T lymphocytes and subsequently classified by CD4 and CD8 expression as helper/inducer T cells (CD3^+^ CD4^+^) or cytotoxic/suppressor T cells (CD3^+^ CD8^+^). results were expressed as both absolute cell counts (cells/μL) and percentages relative to the total lymphocyte population. Composite systemic inflammatory indices were derived from the aforementioned parameters: NLR (NLR = NEU/LYMPH, PLR (PLR = PLT/LYMPH), LMR (LMR = LYMPH/MONO), and SII (SII = NEU × PLT/LYMPH). For patients in the PTB + CNS-TB cohort, CSF was obtained by standard lumbar puncture at admission; CSF parameters included total protein (CSF-PRO, mg/L), glucose (CSF-GLU, mmol/L), chloride (CSF-Cl, mmol/L), total leukocyte count (CSF-WBC, ×10^6^/L), and absolute and proportional mononuclear (CSF-MNC, CSF-MNC%) and polymorphonuclear (CSF-PMN, CSF-PMN%) cell counts. All laboratory tests were performed according to standardized operating procedures, with the initial test results documented in the hospital information system and used for analysis. For the inter-cohort PSM analysis, the primary exposure was disease group status (PTB + CNS-TB vs. PTB). Sex, age, BMI, ALB, and Hb were included to mitigate baseline imbalances and minimize potential confounding of peripheral lymphocyte and T-cell profiles. Specifically, BMI, ALB, and Hb were used jointly to align nutritional status. Due to its longer half-life, ALB further reflected disease duration, chronic consumption, and volume status, thereby improving the comparability of pre-admission baseline status between cohorts. For the intra-cohort severity analysis, the primary exposure was MRC-defined severity (severe [Grades II–III, *n* = 86; including 55 (64.0%) Grade II and 31 (36.0%) Grade III] vs. mild [Grade I, *n* = 165]), and the same five variables were included as adjustment covariates in the multivariable regression model. The clinical dataset was complete for all included patients, as completeness of key laboratory data was a mandatory inclusion criterion; accordingly, the analysis was conducted as a complete-case analysis with no missing data.

### 2.3 Statistical analysis

All statistical analyses were performed using R version 4.3.3. To mitigate selection bias and balance baseline characteristics for the inter-cohort comparison, PSM was implemented using the *MatchIt* package, incorporating sex, age, BMI, ALB, and Hb into a logistic regression propensity score model. Matching was performed using the nearest-neighbor method with a caliper width of 0.2 standard deviations of the logit of the propensity score and a 1:4 matching ratio (set.seed = 2025), informed by methodological evidence that a matching ratio up to 4:1 (control to treatment) generally elicits the lowest bias (Linden and Samuels, 2013) and by the efficiency principle that M/(M + 1) approximates matching efficiency, where 4 controls per case yields ~80% efficiency with negligible gains once M exceeds 5 (Austin, 2010). Of 251 eligible PTB + CNS-TB patients and 4578 eligible PTB controls, 246 PTB + CNS-TB patients were successfully matched to 954 unique PTB controls; 5 PTB + CNS-TB patients were excluded from the matched cohort because no eligible control remained within the caliper. Because matching was performed without replacement under caliper restriction, the realized number of controls per case was not fixed at four for every matched set; the achieved ratio was below 1:4 for a subset of matched cases, accounting for the total of 954 controls rather than the theoretical maximum of 984. Matched-set identifiers (subclass) and matching weights generated by MatchIt were retained for all post-matching analyses. For post-matching comparisons, inference explicitly accounted for the matched design. Continuous outcomes with approximately normal distributions were analyzed using weighted linear regression with CR2 cluster-robust standard errors clustered by matched set. For non-normally distributed continuous outcomes, rank-transformed outcomes were analyzed using the same weighted, matched-set-clustered regression framework. Binary outcomes were analyzed using weighted generalized estimating equations with matched set as the clustering unit. Covariate balance before and after matching was evaluated using standardized mean differences (SMD), calculated as the absolute difference in means divided by the pooled standard deviation for continuous variables, and the absolute difference in proportions divided by the pooled standard deviation for binary variables; SMD < 0.1 was pre-specified as the threshold for adequate balance. SMDs, rather than null-hypothesis P values, were used to evaluate covariate balance after matching. The distribution and balance of propensity scores were visualized using density plots and Love plots, generated with the ggplot2 and cobalt packages.

To address concerns regarding the choice of a 1:4 matching ratio and to confirm the robustness of our findings, we performed a sensitivity analysis using inverse probability of treatment weighting (IPTW). Propensity score weights were estimated by logistic regression with the same five covariates (sex, age, BMI, ALB, and Hb) under the average treatment effect on the treated (ATT) estimand using the *WeightIt* package. Covariate balance was assessed by SMD, and the effective sample size (ESS) was reported. As a sensitivity analysis, IPTW was performed using propensity scores for PTB+CNS-TB group membership estimated from age, sex, BMI, albumin, and hemoglobin. The weighting approach targeted the covariate distribution of the PTB+CNS-TB group, with PTB participants reweighted accordingly. Weights were truncated at the 1st and 99th percentiles. For IPTW balance diagnostics, both unweighted and weighted SMDs were calculated using the standard deviation of the PTB+CNS-TB group as the standardizing denominator, consistent with the ATT estimand. Covariate balance was assessed using absolute standardized mean differences (SMDs), with values <0.1 indicating adequate balance. IPTW-weighted regression models with robust standard errors were used for inference; rank-transformed outcomes were used for non-normally distributed continuous variables. P values were adjusted using the Benjamini–Hochberg false-discovery-rate procedure.

For comparisons in the unmatched cohort, normality of continuous variables was assessed using the Shapiro–Wilk test (applicable range: 3 ≤ n ≤ 5000). Normally distributed variables were expressed as mean ± standard deviation (SD) and compared using the independent-samples t-test, with Levene’s test used to determine whether the equal-variance or Welch-corrected form was applied. Non-normally distributed variables were expressed as median (interquartile range, IQR) and compared using the Wilcoxon rank-sum test with continuity correction. Categorical variables were summarized as frequency and percentage and compared using the chi-square test or Fisher’s exact test, as appropriate. For comparisons in the PSM cohort, the matched-set-clustered, weighted analytical procedures described above were used instead of independent-sample t-tests, Wilcoxon rank-sum tests, chi-square tests, or Fisher’s exact tests. SMD values were reported alongside descriptive statistics. Between-group distributions were visualized as raincloud plots.

For the intra-cohort severity analysis, the association between each laboratory parameter and MRC severity group was evaluated by covariate-adjusted regression (adjusted for age, sex, BMI, ALB, and Hb). For each outcome variable, normality was assessed using the Shapiro–Wilk test: normally distributed outcomes were analyzed by ordinary least-squares linear regression (equivalent to analysis of covariance with continuous covariates), whereas non-normally distributed outcomes were analyzed by rank-based regression, in which the outcome variable was rank-transformed prior to model fitting. Standardized beta coefficients (Std.β) were derived from models in which all continuous variables, including outcomes and continuous covariates, were z-score standardized prior to fitting. Multiple testing was addressed using the Benjamini–Hochberg FDR correction (*p.adjust*, method = “BH”) across all tested outcomes; statistical significance was defined as FDR-adjusted *P* < 0.05. Results were visualized as a forest plot of standardized beta coefficients with 95% confidence intervals, constructed using *ggplot2*, with color coding according to FDR-adjusted significance. Severity-stratified distributions of key peripheral and CSF parameters were additionally displayed as boxplots for Mild and Severe groups.

Independently associated factors were subsequently identified from parameters showing collinearity: candidates were grouped by hierarchical clustering (neutrophil-inflammation, CSF-inflammation, T-lymphocyte axes), each represented by one variable (NEU% and CSF protein pre-specified on biological grounds), then screened by LASSO regression (10-fold cross-validation) and refined with Firth’s bias-corrected logistic regression, yielding NEU%, CSF protein, and ALB as independent factors. These three factors were combined into a severity assessment model and evaluated by ROC analysis, with pairwise AUC comparisons against individual markers using the DeLong test (Bonferroni-corrected). Incremental value over CSF protein alone was quantified by continuous and categorical NRI and IDI (bootstrap-based P values), with calibration assessed via the Hosmer–Lemeshow test and calibration slope, and internal validation via bootstrap resampling (B = 500) for optimism-corrected AUC. A sensitivity model additionally adjusting for age, sex, BMI, and Hb was compared using the DeLong test.

## 3 Results

### 3.1 Balancing baseline characteristics through PSM: PTB + CNS-TB vs. PTB cohorts

Among the 251 patients in the PTB + CNS-TB group, 105 (41.83%) were microbiologically confirmed, and 146 (58.17%) were clinically diagnosed. To mitigate confounding effects, PSM was applied to the PTB + CNS-TB group (*n* = 251) and the PTB group (*n* = 4578), yielding a matched cohort of 246 PTB + CNS-TB patients and 954 PTB controls. The propensity score model included sex, age, BMI, ALB, and Hb. Before matching, the PTB + CNS-TB group had lower BMI, ALB, and Hb than the PTB group, consistent with poorer baseline nutritional status. After matching, all five prespecified matching covariates achieved adequate balance, with absolute SMDs < 0.1 (**Supplementary Figure 1**). Specifically, the post-matching absolute SMDs were 0.033 for age, 0.001 for sex, 0.053 for BMI, 0.039 for ALB, and 0.037 for Hb. The propensity-score density plot demonstrated substantial overlap between the matched groups, and the Love plot confirmed improved covariate balance after matching (**Supplementary Figures 1**, **2**). Balance was evaluated using SMDs rather than hypothesis-test *P* values. In the IPTW sensitivity analysis, covariate balance was adequate for all prespecified weighting covariates, with absolute weighted SMDs ranging from 0.008 to 0.017 (**Supplementary Table 2**). The effective sample size of the weighted PTB group was 2,027 (44.3% of the original PTB sample), indicating acceptable weight stability (**Supplementary Table 3**). The IPTW analysis yielded results consistent with the PSM analysis, including higher neutrophil-related indices and lower lymphocyte and T-cell counts in the PTB + CNS-TB group (**Supplementary Table 4**).

To characterize the representativeness of the matched PTB control group, we compared clinical and laboratory profiles between matched (*n* = 954) and unmatched (*n* = 3,624) PTB controls (**Supplementary Table 5**). The matched PTB controls exhibited significantly lower BMI (19.53 vs. 20.55 kg/m², *P* < 0.001), albumin (37.00 vs. 40.30 g/L, *P* < 0.001), and hemoglobin (116.00 vs. 130.00 g/L, *P* < 0.001), alongside higher NLR (3.46 vs. 2.60, *P* < 0.001), PLR (208.68 vs. 159.82, *P* < 0.001), SII (962.21 vs. 627.12, *P* < 0.001), and lower lymphocyte count (1.35 vs. 1.54 ×10^9^/L, *P* < 0.001) and CD4^+^ T cell count (601.50 vs. 672.50 cells/μL, *P* < 0.001) than unmatched controls.

### 3.2 Distinctive immuno-inflammatory profile in PTB + CNS-TB patients after PSM

**Figure 2:**
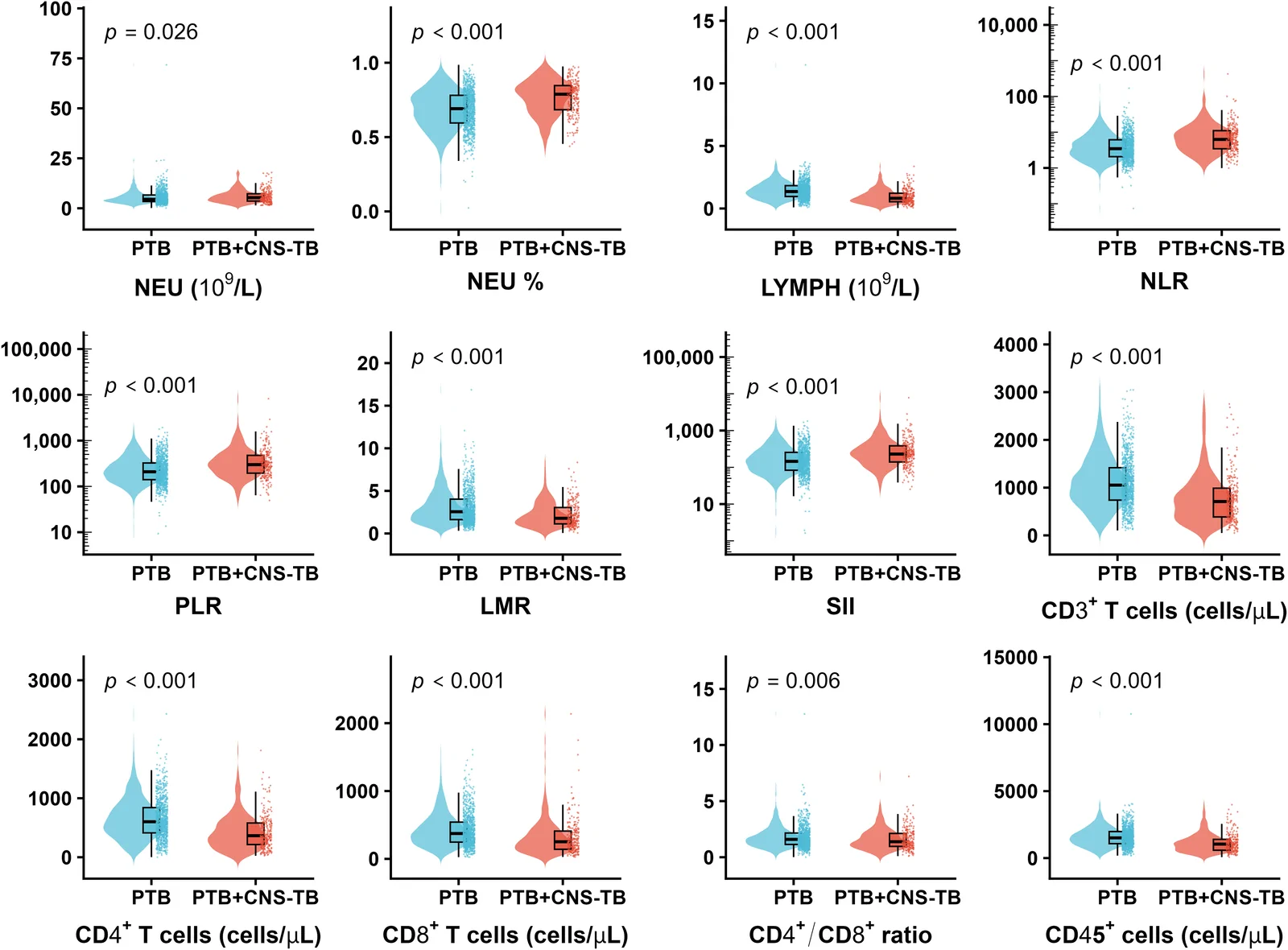
Raincloud plots comparing immune and inflammatory biomarkers between PTB and PTB + CNS-TB groups. Each panel displays the distribution of the indicated parameter in the propensity score–matched cohort for patients with pulmonary tuberculosis alone (PTB, blue) and those with concurrent central nervous system tuberculosis (PTB+CNS-TB, red). The cloud (half-violin) represents the kernel density estimate; the box indicates the interquartile range (IQR) with the median line; and dots represent individual data points, jittered to reduce overplotting. NLR, PLR, and SII are displayed on a logarithmic scale. P values were derived from regression models incorporating matching weights, with CR2 cluster-robust standard errors clustered by matched set (subclass); non-normally distributed continuous variables were rank-transformed before analysis. All P values are two-sided and nominal (not adjusted for multiple comparisons). CD45^+^ cells indicates total lymphocytes (cells/μL).

Despite improved balance in the prespecified demographic and nutritional covariates, multiple inflammatory and immunological parameters remained different between the matched groups, revealing a distinctive immuno-inflammatory profile in PTB + CNS-TB patients (**Table 1**, **Figure 2**). All post-matching P values reported in **Table 1** and **Figure 2** were obtained from weighted analyses that accounted for clustering within matched sets.

**Table 1:** Clinical parameters of patients with PTB + CNS-TB and PTB after propensity score matching.

Variable	PTB+CNS-TB (*n* = 246)	PTB (*n* = 954)	SMD	Statistic	*P* value
Sex (Male), n (%)	166 (67.5%)	644 (67.5%)	0.001	Wald *χ²* = 0.006	
Age,M(Q_1_,Q_3_)	56.50 (37.00,67.00)	56.00 (39.00,68.00)	0.033	*t* = -0.369	0.712
BMI,M(Q_1_,Q_3_)	19.24 (17.88,21.44)	19.53 (17.72,21.88)	0.053	*t* = -0.428	0.669
ALB,M(Q_1_,Q_3_)	36.05 (32.37,39.69)	37.00 (32.83,39.90)	0.039	*t* = -0.805	0.422
Hb,M(Q_1_,Q_3_)	115.00 (101.00,126.00)	116.00 (104.00,129.00)	0.037	*t* = -0.278	0.781
WBC,M(Q_1_,Q_3_)	6.83 (5.10,8.86)	6.79 (5.57,8.77)	0.049	*t* = -0.838	0.403
NEU,M(Q_1_,Q_3_)	5.32 (3.60,7.25)	4.51 (3.41,6.62)	0.132	*t* = 2.233	0.026
NEU%,M(Q_1_,Q_3_)	0.79 (0.68,0.85)	0.69 (0.59,0.78)	0.680	*t* = 9.578	< 0.001
LYMPH,M(Q_1_,Q_3_)	0.81 (0.54,1.22)	1.35 (0.96,1.83)	0.757	*t* = -11.843	< 0.001
MONO,M(Q_1_,Q_3_)	0.46 (0.32,0.65)	0.51 (0.37,0.67)	0.179	*t* = -2.559	0.011
RBC,M(Q_1_,Q_3_)	4.03 (3.62,4.45)	4.09 (3.70,4.50)	0.089	*t* = -0.162	0.872
PLT,M(Q_1_,Q_3_)	249.50 (195.25,319.75)	279.50 (225.00,359.00)	0.318	*t* = -4.493	< 0.001
NLR,M(Q_1_,Q_3_)	6.28 (3.45,11.04)	3.46 (2.07,6.14)	0.280	*t* = 9.520	< 0.001
PLR,M(Q_1_,Q_3_)	298.85 (194.64,475.95)	208.68 (140.50,324.43)	0.338	*t* = 7.613	< 0.001
LMR,M(Q_1_,Q_3_)	1.78 (1.13,3.05)	2.55 (1.63,4.03)	0.498	*t* = -6.494	< 0.001
SII,M(Q_1_,Q_3_)	1611.02 (830.95,2761.89)	962.21 (508.91,2062.33)	0.206	*t* = 6.092	< 0.001
CD3^+^%,M(Q_1_,Q_3_)	69.50 (60.25,76.00)	72.00 (64.00,77.00)	0.207	*t* = -2.514	0.013
CD3^+^,M(Q_1_,Q_3_)	708.50 (386.00,988.50)	1052.50 (738.25,1417.75)	0.664	*t* = -9.804	< 0.001
CD4^+^%,M(Q_1_,Q_3_)	37.00 (30.00,45.00)	41.00 (34.00,47.00)	0.333	*t* = -4.410	< 0.001
CD4^+^,M(Q_1_,Q_3_)	364.50 (215.50,579.75)	601.50 (413.00,841.00)	0.713	*t* = -10.478	< 0.001
CD8^+^%,M(Q_1_,Q_3_)	27.50 (20.00,35.00)	26.00 (20.00,32.00)	0.106	*t* = 1.119	0.264
CD8^+^,M(Q_1_,Q_3_)	252.50 (141.75,409.50)	374.00 (247.00,541.50)	0.411	*t* = -7.487	< 0.001
CD4^+^/CD8^+^,M(Q_1_,Q_3_)	1.38 (0.96,2.12)	1.59 (1.14,2.16)	0.152	*t* = -2.800	0.006
CD45^+^,M(Q_1_,Q_3_)	1043.00 (585.25,1398.50)	1508.50 (1078.50,1980.75)	0.636	*t* = -9.874	< 0.001

PTB, pulmonary tuberculosis; CNS-TB, central nervous system tuberculosis; PSM, propensity score matching; BMI, body mass index; ALB, albumin (g/L); Hb, hemoglobin (g/L); WBC, white blood cell (×10^9^/L); NEU, neutrophil (×10^9^/L); NEU%, neutrophil percentage; LYMPH, lymphocyte (×10^9^/L); MONO, monocyte (×10^9^/L); RBC, red blood cell (×10^12^/L); PLT, platelet (×10^9^/L); NLR, neutrophil-to-lymphocyte ratio; PLR, platelet-to-lymphocyte ratio; LMR, lymphocyte-to-monocyte ratio; SII, systemic immune-inflammation index (calculated from neutrophil, platelet, and lymphocyte counts). Lymphocyte subset analysis: CD3^+^ indicates total T lymphocytes (cells/μL, %); CD3^+^% indicates the percentage of CD3^+^ T lymphocytes relative to total lymphocyte population; CD4^+^ indicates helper T cells (cells/μL, %); CD4^+^% indicates the percentage of CD4^+^ T lymphocytes relative to total lymphocyte population; CD8^+^ indicates cytotoxic T lymphocytes (cells/μL, %); CD8^+^% indicates the percentage of CD8^+^ T lymphocytes relative to total lymphocyte population; CD4^+^/CD8^+^ indicates the ratio of CD4^+^ to CD8^+^ T lymphocytes; CD45^+^ indicates total lymphocytes (cells/μL). SMD, standardized mean difference. Data are presented as median (interquartile range, IQR) or *n* (%). Propensity score matching was performed using 1:4 nearest-neighbor matching without replacement, with a caliper width of 0.2 SD of the logit of the propensity score. Matching covariates were age, sex, BMI, albumin, and hemoglobin. Covariate balance was assessed using absolute standardized mean differences (SMDs), with an absolute SMD < 0.1 indicating adequate balance. Post-matching P values accounted for matching weights and clustering within matched sets (subclass). Continuous outcomes were analyzed using weighted linear regression with CR2 cluster-robust standard errors; non-normally distributed continuous outcomes were rank-transformed before analysis. Binary outcomes were analyzed using weighted generalized estimating equations clustered by matched set.

#### 3.2.1 Elevated systemic inflammatory markers

After PSM, PTB + CNS-TB patients demonstrated significantly higher neutrophil counts compared to PTB patients (5.32 vs. 4.51 ×10^9^/L, *t* = 2.233, *P* = 0.026, SMD = 0.132). The NEU% was substantially elevated in the PTB + CNS-TB group (0.79 vs. 0.69, *t* = 9.578, *P* < 0.001, SMD = 0.680), representing one of the most pronounced inter-group differences observed. Composite inflammatory indices were consistently elevated in PTB + CNS-TB patients: the NLR was markedly higher (6.28 vs. 3.46, *W* = 161143.5, *P* < 0.001, SMD = 0.280), as was the PLR (298.85 vs. 208.68, *t* = 7.613, *P* < 0.001, SMD = 0.338) and the SII (1611.02 vs. 962.21, *t* = 6.092, *P* < 0.001, SMD = 0.206).

#### 3.2.2 Reduced peripheral lymphocyte and T-cell counts in PTB + CNS-TB patients

Conversely, LYMPH counts were profoundly reduced in PTB + CNS-TB patients relative to matched PTB controls (0.81 vs. 1.35 ×10^9^/L, *t* = -11.843, *P* < 0.001, SMD = 0.757), representing the largest standardized mean difference observed across all post-matching comparisons. The LMR was correspondingly lower in the PTB + CNS-TB group (1.78 vs. 2.55, *t* = -6.494, *P* < 0.001, SMD = 0.498). Analysis of T lymphocyte subsets revealed that absolute counts of CD3^+^ (708.50 vs. 1052.50 cells/μL, *t* = -9.804, *P* < 0.001, SMD = 0.664), CD4^+^ (364.50 vs. 601.50 cells/μL, *t* = -10.478, *P* < 0.001, SMD = 0.713), and CD8^+^ (252.50 vs. 374.00 cells/μL, *t* = -7.487, *P* < 0.001, SMD = 0.411) T lymphocytes were all significantly lower in the PTB + CNS-TB group. In addition, the CD45^+^ lymphocyte count (1043.00 vs. 1508.50 cells/μL, *t* = -9.874, *P* < 0.001, SMD = 0.636) was lower in the PTB + CNS-TB group. The percentages of CD3^+^ and CD4^+^ T cells were also significantly reduced (CD3^+^%: 69.50 vs. 72.00, *P* = 0.013, SMD = 0.207; CD4^+^%: 37.00 vs. 41.00, *P* < 0.001, SMD = 0.333).

### 3.3 Comparative clinical and laboratory profiles between mild and severe PTB + CNS-TB patients

Among the 251 PTB + CNS-TB patients, 165 (65.7%) were classified as mild and 86 (34.3%) as severe. Baseline demographic characteristics, including sex (P = 0.462), age (P = 0.966), and BMI (P = 0.909), were comparable between the two groups. (**Table 2**).

**Table 2:** Baseline characteristics and laboratory parameters of mild and severe PTB + CNS-TB patients.

Variable	Mild group(*n* = 165)	Severe group(*n* = 86)	Statistic	*P*	SMD
Sex, *n*(%)			*χ²* = 0.542	0.462	0.118
Male	108(65.45)	61(70.93)			
Female	57(34.54)	25(29.07)			
Age, M(Q_1_,Q_3_)	57.00(35.00,67.00)	56.00(39.00,66.75)	*W* = 7071.5	0.966	0.022
BMI,M(Q_1_,Q_3_)	19.27(17.72,21.22)	18.78(17.88,21.45)	*W* = 7158.0	0.909	0.030
ALB, Mean ± SD	35.15 ± 5.80	37.51 ± 4.63	*t* = -3.505	< 0.001	0.435
Hb, Mean ± SD	110.87 ± 20.72	118.98 ± 18.40	*t* = -3.056	0.002	0.406
WBC,M(Q_1_,Q_3_)	6.34(4.79,8.38)	7.84(5.96,9.67)	*W* = 5190.0	< 0.001	0.413
NEU,M(Q_1_,Q_3_)	4.69(3.35,6.60)	6.19(4.81,8.07)	*W* = 4972.5	< 0.001	0.464
NEU%,M(Q_1_,Q_3_)	0.76(0.68,0.83)	0.82(0.74,0.87)	*W* = 5149.5	< 0.001	0.479
LYMPH,M(Q_1_,Q_3_)	0.83(0.54,1.22)	0.78(0.54,1.18)	*W* = 7537.0	0.419	0.066
MONO,M(Q_1_,Q_3_)	0.45(0.31,0.68)	0.48(0.34,0.62)	*W* = 6911.5	0.737	0.001
RBC,M(Q_1_,Q_3_)	3.99(3.44,4.41)	4.08(3.80,4.50)	*W* = 6100.0	0.068	0.088
PLT,M(Q_1_,Q_3_)	263.00(200.00,330.00)	230.50(182.50,295.25)	*W* = 8275.5	0.031	0.279
PLR,M(Q_1_,Q_3_)	313.58(193.86,487.50)	294.02(199.23,440.20)	*W* = 7294.5	0.715	0.146
NLR,M(Q_1_,Q_3_)	5.75(3.28,9.41)	8.83(4.43,13.13)	*W* = 5431.0	0.002	0.278
LMR,M(Q_1_,Q_3_)	1.81(1.17,3.06)	1.58(1.02,2.82)	*W* = 7603.0	0.353	0.111
SII,M(Q_1_,Q_3_)	1381.33 (747.80, 2663.11)	1879.56 (1099.79, 3092.12)	*W* = 6009.0	0.047	0.223
CD3^+^,M(Q_1_,Q_3_)	682.00(384.00,991.00)	725.50(367.75,956.75)	*W* = 7121.0	0.963	0.009
CD3^+^%,M(Q_1_,Q_3_)	71.00(62.00,77.00)	68.00(59.00,74.00)	*W* = 8004.5	0.096	0.286
CD4^+^,M(Q_1_,Q_3_)	354.00(221.00,560.00)	380.50(204.75,582.00)	*W* = 6900.5	0.722	0.113
CD4^+^%,Mean ± SD	37.29 ± 11.11	37.06 ± 11.95	*t* = 0.153	0.878	0.020
CD8^+^,M(Q_1_,Q_3_)	244.00(146.00,412.00)	255.50(123.25,386.75)	*W* = 7436.5	0.532	0.131
CD8^+^%,M(Q_1_,Q_3_)	28.00(21.00,35.00)	26.00(19.00,34.75)	*W* = 8022.0	0.089	0.299
CD4^+^/CD8^+^,M(Q_1_,Q_3_)	1.38(0.96,2.05)	1.44(0.97,2.18)	*W* = 6674.5	0.442	0.179
CD45^+^,M(Q_1_,Q_3_)	983.00(537.00,1394.00)	1054.00(639.50,1381.25)	*W* = 6856.0	0.662	0.013
CSF-PRO,M(Q_1_,Q_3_)	716.40(417.00,1295.60)	1501.60(797.58,2689.25)	*W* = 3987.0	< 0.001	0.560
CSF-Cl,M(Q_1_,Q_3_)	120.80(114.80,123.30)	117.05(111.38,121.00)	*W* = 9016.5	< 0.001	0.511
CSF-GLU,M(Q_1_,Q_3_)	3.08(2.56,3.67)	2.86(2.19,4.04)	*W* = 7850.5	0.167	0.050
CSF-WBC,M(Q_1_,Q_3_)	7.01(2.00,62.01)	74.50(14.76,161.26)	*W* = 4250.0	< 0.001	0.233
CSF-MNC%,M(Q_1_,Q_3_)	0.81(0.50,0.97)	0.86(0.58,0.97)	*W* = 6696.5	0.464	0.210
CSF-MNC,M(Q_1_,Q_3_)	4.01(1.01,44.01)	50.00(10.26,112.00)	*W* = 4290.0	< 0.001	0.395
CSF-PMN%,M(Q_1_,Q_3_)	0.20(0.03,0.50)	0.14(0.03,0.42)	*W* = 7574.0	0.379	0.229
CSF-PMN,M(Q_1_,Q_3_)	1.34(1.00,8.01)	5.53(1.01,45.76)	*W* = 5169.5	< 0.001	0.002

Mild group, Grades I; Severe group, Grades II–III; Cerebrospinal fluid (CSF) parameters: CSF-PRO, CSF protein (mg/L); CSF-Cl, CSF chloride (mmol/L); CSF-GLU, CSF glucose (mmol/L); CSF-WBC, CSF white blood cell count (×10^6^/L); CSF-MNC%, CSF mononuclear cell percentage; CSF-MNC, CSF mononuclear cell count (×10^6^/L); CSF-PMN%, CSF polymorphonuclear cell percentage; CSF-PMN, CSF polymorphonuclear cell count (×10^6^/L).

#### 3.3.1 Divergent hematological and inflammatory profiles

**Figure 3:**
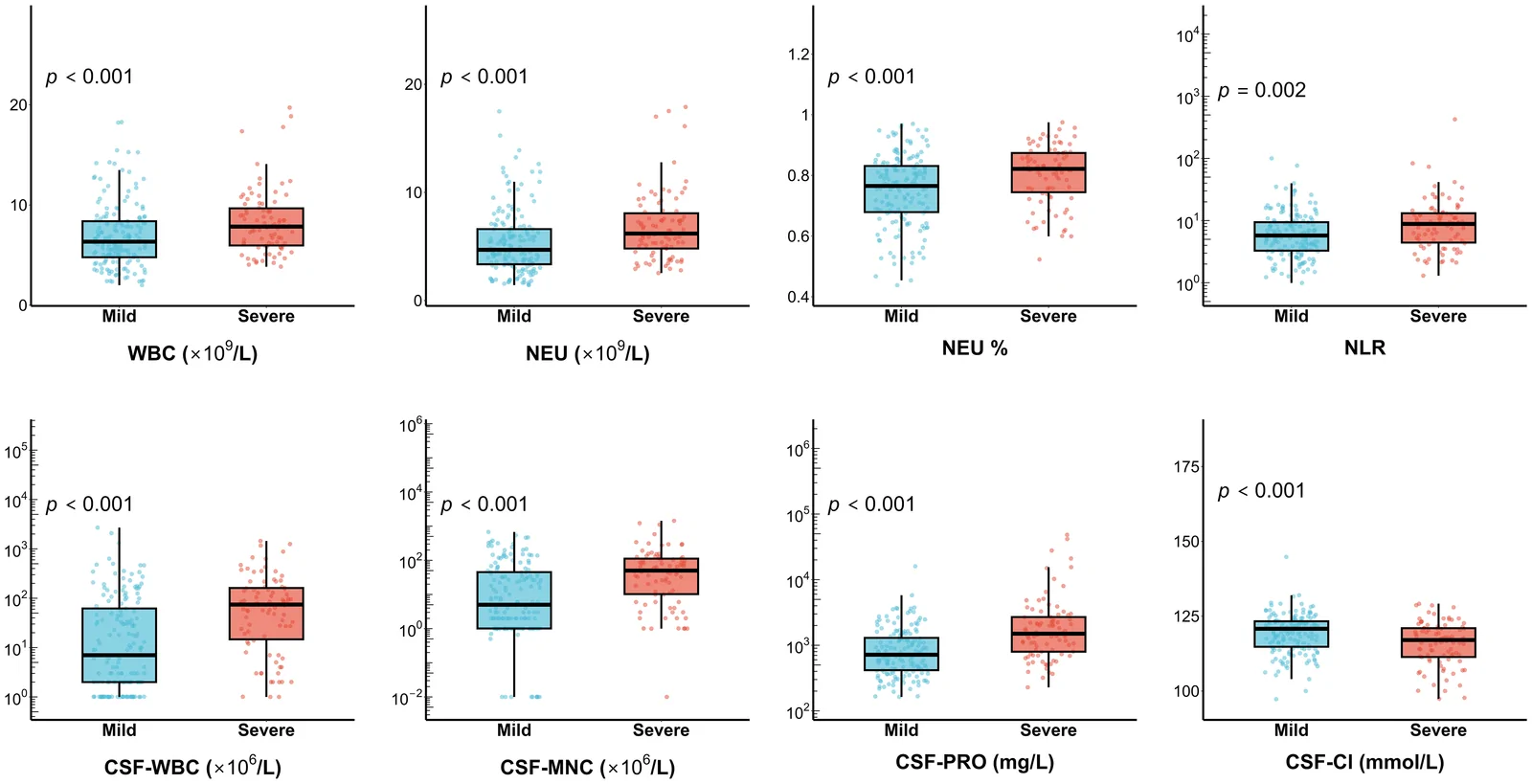
Box plots comparing peripheral blood and cerebrospinal fluid biomarkers between mild and severe CNS-TB groups. Each panel displays the distribution of the indicated biomarker for patients classified as mild (blue) or severe (red) CNS-TB. The central line within each box represents the median; the lower and upper boundaries of the box indicate the 25th and 75th percentiles (interquartile range, IQR); whiskers extend to 1.5 × IQR; individual data points are shown as jittered dots. Variables with skewed distributions (NLR, CSF-WBC, CSF-MNC, and CSF-PRO) are presented on a logarithmic scale with corresponding log-tick marks on the y-axis. *P* values are unadjusted group comparison *P* values.

The severe group exhibited a markedly distinct hematological profile (**Table 2**, **Figure 3**). ALB (37.51 ± 4.63 vs. 35.15 ± 5.80 g/L, *t* = −3.505, *P* < 0.001, SMD = 0.435) and Hb (118.98 ± 18.40 vs. 110.87 ± 20.72 g/L, *t* = −3.056, *P* = 0.002, SMD = 0.406) were significantly higher in the severe group. Prominent neutrophilia was observed in the severe group, with significantly elevated WBC [7.84 (5.96, 9.67) vs. 6.34 (4.79, 8.38) ×10^9^/L, *W* = 5190.0, *P* < 0.001], absolute neutrophil counts [6.19 (4.81, 8.07) vs. 4.69 (3.35, 6.60) ×10^9^/L, *W* = 4972.5, *P* < 0.001], and NEU% [0.82 (0.74, 0.87) vs. 0.76 (0.68, 0.83), *W* = 5149.5, P < 0.001]. The NLR was significantly elevated in the severe group [8.83 (4.43, 13.13) vs. 5.75 (3.28, 9.41), *W* = 5431.0, *P* = 0.002, SMD = 0.278], while PLT was significantly lower [230.50 (182.50, 295.25) vs. 263.00 (200.00, 330.00) ×10^9^/L, *W* = 8275.5, *P* = 0.031]. PLR, LMR, and SII showed no significant between-group differences (all *P* > 0.05). Notably, peripheral T-lymphocyte subsets (including CD3^+^, CD4^+^, and CD8^+^ counts and percentages) and CD45^+^ lymphocyte parameters were comparable between the two groups (all P > 0.05).

#### 3.3.2 Markedly elevated intrathecal inflammatory burden in severe patients

The most clinically discriminating differences resided in CSF parameters (**Table 2**, **Figure 3**). CSF protein was dramatically higher in the severe group [1,501.60 (797.58, 2,689.25) vs. 716.40 (417.00, 1,295.60) mg/L, *W* = 3987.0, *P* < 0.001, SMD = 0.560], while CSF chloride was significantly reduced [117.05 (111.38, 121.00) vs. 120.80 (114.80, 123.30) mmol/L, *W* = 9016.5, *P* < 0.001, SMD = 0.511]. CSF-WBC [74.50 (14.76, 161.26) vs. 7.01 (2.00, 62.01) ×10^6^/L, *W* = 4250.0, *P* < 0.001], absolute mononuclear cell counts [50.00 (10.26, 112.00) vs. 4.01 (1.01, 44.01) ×10^6^/L, *W* = 4290.0, *P* < 0.001, SMD = 0.395], and polymorphonuclear cell counts [5.53 (1.01, 45.76) vs. 1.34 (1.00, 8.01) ×10^6^/L, *W* = 5169.5, *P* < 0.001] were all substantially elevated in the severe group, whereas the proportional cellular composition of CSF did not significantly differ (both *P* > 0.05).

#### 3.3.3 Covariate-adjusted association between laboratory parameters and severity

**Figure 4:**
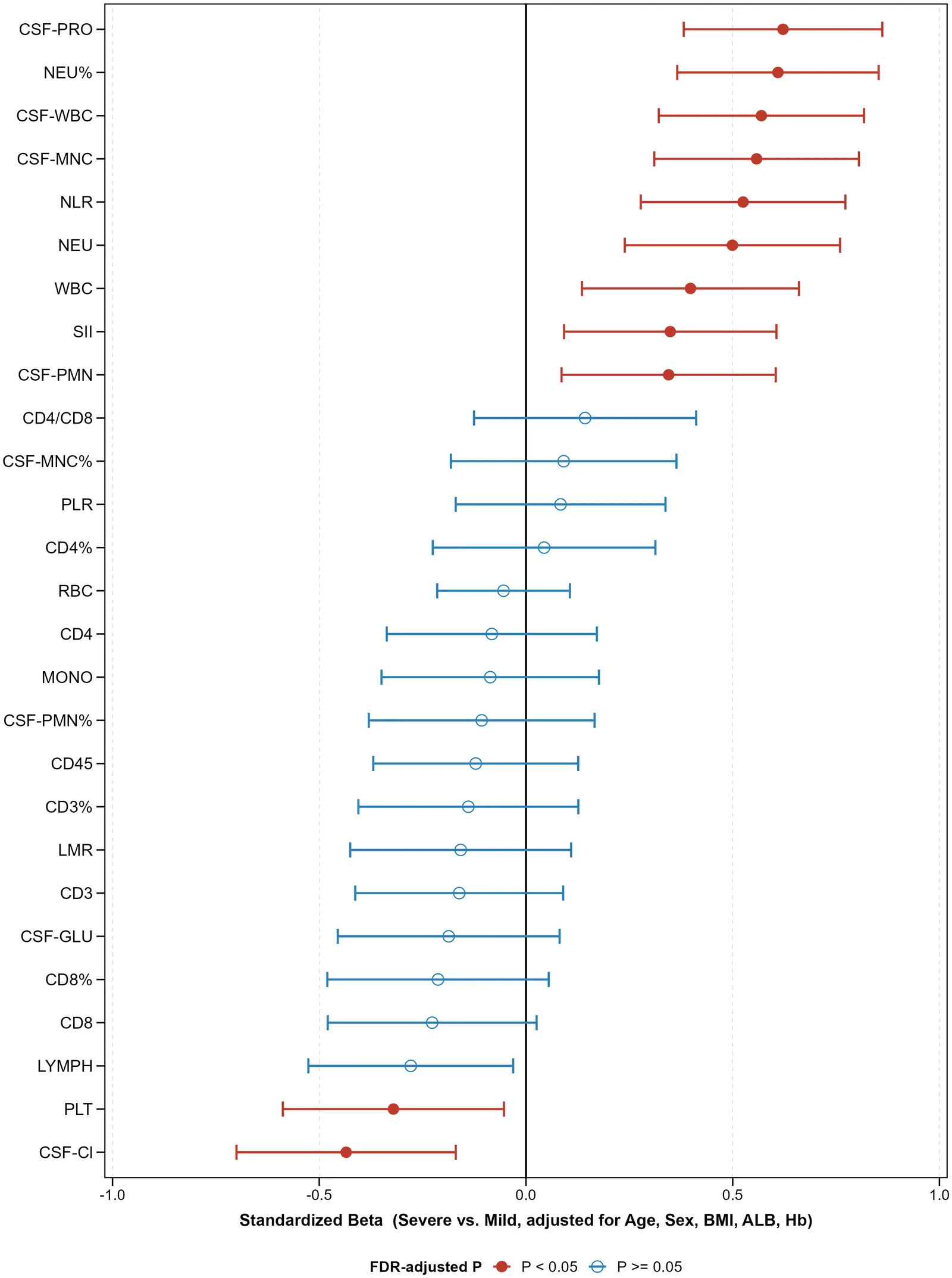
Forest plot of standardized beta coefficients for clinical indicators associated with the severity of PTB + CNS-TB. FDR, false discovery rate. Filled squares indicate statistically significant indicators after Benjamini–Hochberg FDR correction (*P* < 0.05); dashed line represents nonsignificant results.

To assess the association between individual laboratory parameters and disease severity while accounting for baseline demographic and nutritional differences, covariate-adjusted regression models were performed for each parameter separately, with adjustment for age, sex, BMI, ALB, and Hb (**Table 3**, **Figure 4**). It should be noted that, because several systemic inflammatory indices (e.g., NEU%, NLR, NEU, WBC) are mathematically or biologically correlated, each parameter was evaluated in a separate adjusted model rather than a single joint multivariable model; therefore, the estimates reflect covariate-adjusted associations rather than mutually independent effects among these correlated indices.

**Table 3:** Multivariable regression analysis of laboratory parameters associated with PTB + CNS-TB severity (adjusted for age, sex, BMI, ALB, and Hb).

Variable	*β*	SE	95% CI	*t*	*P*	FDR *P*	Std.*β*
WBC	28.885	9.67	(9.837, 47.932)	2.987	0.003	0.010	0.398
NEU	36.247	9.597	(17.344, 55.15)	3.777	< 0.001	< 0.001	0.499
NEU%	44.244	8.978	(26.56, 61.928)	4.928	< 0.001	< 0.001	0.609
LYMPH	-20.228	9.126	(-38.205, -2.252)	-2.216	0.028	0.068	-0.279
MONO	-6.279	9.691	(-25.367, 12.809)	-0.648	0.518	0.543	-0.086
RBC	-3.94	5.915	(-15.59, 7.71)	-0.666	0.506	0.543	-0.054
PLT	-23.281	9.859	(-42.702, -3.861)	-2.361	0.019	0.051	-0.321
PLR	6.076	9.352	(-12.345, 24.497)	0.65	0.516	0.543	0.084
NLR	38.12	9.12	(20.156, 56.084)	4.18	< 0.001	< 0.001	0.525
LMR	-11.47	9.846	(-30.865, 7.925)	-1.165	0.245	0.389	-0.158
SII	25.334	9.467	(6.686,43.982)	2.676	0.008	0.240	0.349
CD3^+^%	-10.113	9.798	(-29.414, 9.187)	-1.032	0.303	0.431	-0.139
CD3^+^	-11.727	9.268	(-29.983, 6.528)	-1.265	0.207	0.349	-0.162
CD4^+^%	0.499	1.556	(-2.566, 3.563)	0.321	0.749	0.749	0.044
CD4^+^	-5.999	9.368	(-24.451, 12.452)	-0.64	0.522	0.543	-0.083
CD8^+^%	-15.436	9.864	(-34.865, 3.993)	-1.565	0.119	0.240	-0.213
CD8^+^	-16.476	9.311	(-34.816, 1.863)	-1.77	0.078	0.176	-0.227
CD4^+^/CD8^+^	10.379	9.904	(-9.129, 29.887)	1.048	0.296	0.431	0.143
CD45^+^	-8.824	9.134	(-26.816, 9.169)	-0.966	0.335	0.452	-0.122
CSF-PRO	45.136	8.85	(27.704, 62.568)	5.1	< 0.001	< 0.001	0.622
CSF-Cl	-31.582	9.775	(-50.835, -12.329)	-3.231	0.001	0.005	-0.435
CSF-GLU	-13.576	9.888	(-33.053, 5.902)	-1.373	0.171	0.308	-0.187
CSF-WBC	41.305	9.143	(23.296, 59.314)	4.518	< 0.001	< 0.001	0.569
CSF-MNC%	6.595	10.022	(-13.145, 26.335)	0.658	0.511	0.543	0.091
CSF-MNC	40.456	9.112	(22.508, 58.404)	4.44	< 0.001	< 0.001	0.558
CSF-PMN%	-7.755	10.032	(-27.515, 12.006)	-0.773	0.440	0.543	-0.107
CSF-PMN	24.962	9.515	(6.22, 43.703)	2.623	0.009	0.028	0.345

95% CI, 95% confidence interval; Std.*β*, standardized regression coefficient; SE, standard error; FDR, false discovery rate (Benjamini–Hochberg method). The multivariable model was adjusted for age, sex, BMI, ALB, and Hb.

After FDR correction, both systemic and intrathecal inflammatory parameters showed significant associations with severity. Among systemic indices, NEU% (*β* = 44.244, 95% CI: 26.56–61.93, Std.*β* = 0.609, FDR *P* < 0.001), NLR (Std.*β* = 0.525, FDR *P* < 0.001), NEU (Std.*β* = 0.499, FDR *P* < 0.001), and WBC (Std*.β* = 0.398, FDR *P* = 0.010) were positively associated with severity. Among CSF parameters, CSF-PRO (Std.*β* = 0.622), CSF-WBC (Std.*β* = 0.569), CSF-MNC (Std.*β* = 0.558), and CSF-PMN (Std.*β* = 0.345) were positively associated with severity (all FDR *P* < 0.05), while CSF-Cl was inversely associated (*β* = −31.582, Std.*β* = −0.435, FDR *P* = 0.005). In contrast, all peripheral T lymphocyte subset parameters did not reach statistical significance after FDR correction (all FDR *P* > 0.05).

#### 3.3.4 Collinearity diagnosis and independently associated factors identified by multivariable analysis

To address the collinearity among neutrophil-related indices such as NEU%, NLR, NEU, and WBC, Spearman correlation analysis and hierarchical clustering were performed first (**Supplementary Figures 3**, **4**). WBC, NEU, NEU%, and NLR showed strong pairwise correlations (*r* = 0.44 to 0.96) and clustered into the same branch, indicating that these indices largely capture the same neutrophilic inflammatory signal rather than independent information. Among CSF parameters, CSF-WBC, CSF-MNC, CSF-PMN, CSF-PRO, and CSF-Cl also formed a cluster, with CSF-Cl showing a negative correlation with the other CSF indices. ALB and Hb showed a weaker correlation (*r* = 0.51) and formed a separate branch, distinct from the other indices.

**Figure 5:**
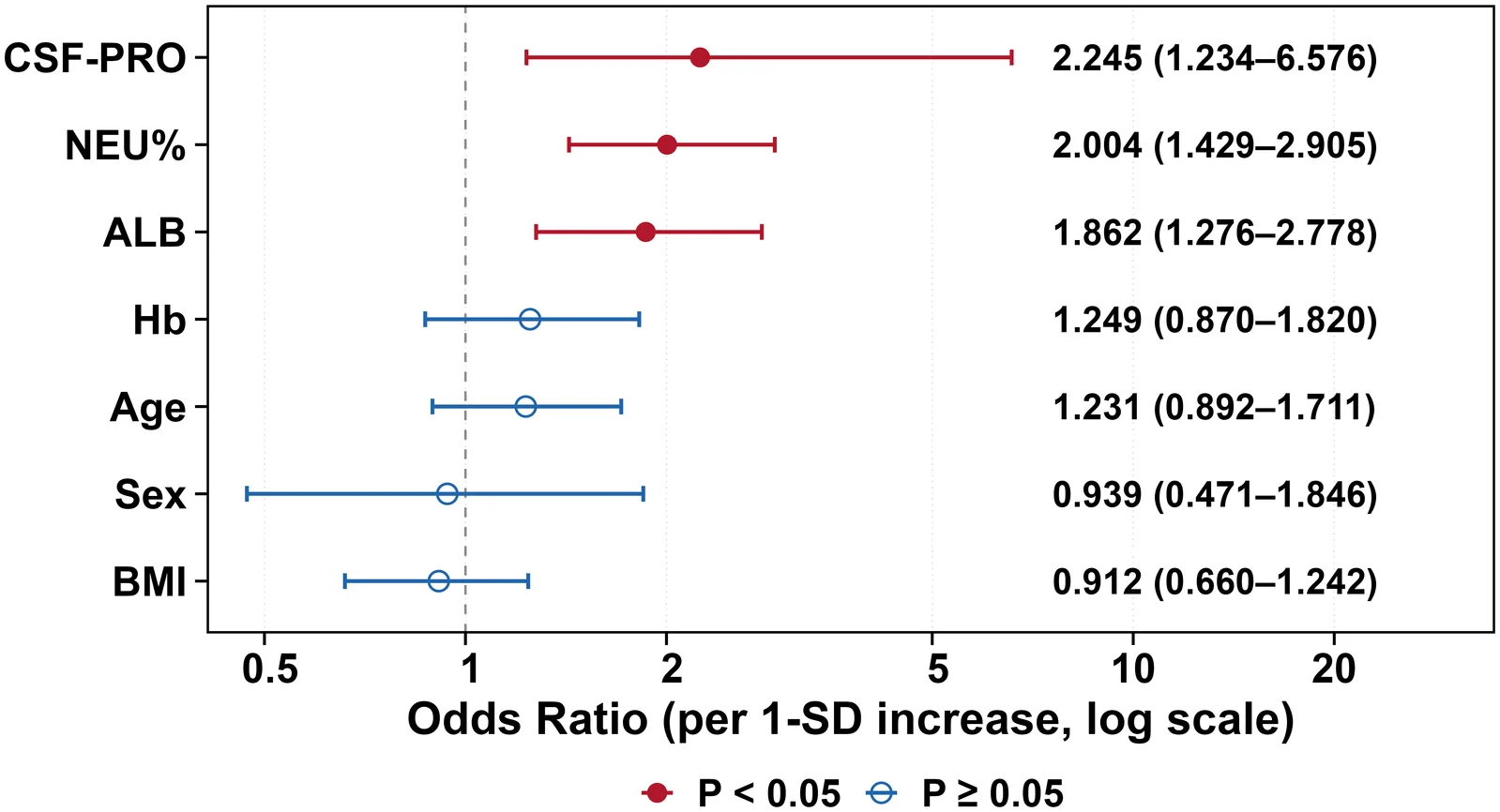
Independent associations of laboratory parameters with severe disease in patients with PTB + CNS-TB. OR, odds ratios. Forest plot of Firth bias-corrected logistic regression results for severity (Severe vs. Mild), showing OR per 1-SD increase for each candidate variable after adjustment for the other variables in the model. Points represent OR estimates and horizontal lines represent 95% CI, plotted on a log scale. Filled red circles indicate P < 0.05; open blue circles indicate P ≥ 0.05. Numeric values to the right of each line show OR (95% CI). The vertical dashed line marks OR = 1.

Based on this collinearity structure, one representative indicator from each correlated cluster was retained for further variable selection, to avoid unstable estimates caused by entering highly correlated indices into the same model. LASSO regression with cross-validation was then used to further narrow down the candidate variables (**Supplementary Figure 5**), followed by Firth’s bias-corrected logistic regression for the final multivariable analysis (**Figure 5**). The variables retained in the final model were CSF-PRO, NEU%, and ALB, together with age, sex, BMI, and Hb as covariates.

The multivariable analysis showed that CSF-PRO, NEU%, and ALB were independently associated with disease severity. Each one-SD increase in CSF-PRO was associated with an odds ratio of 2.245 (95% CI: 1.234–6.576) for severe disease. Each one-SD increase in NEU% corresponded to an odds ratio of 2.004 (95% CI: 1.429 –2.905), and each one-SD increase in ALB corresponded to an odds ratio of 1.862 (95% CI: 1.276– 2.778).

Hb, age, sex, and BMI did not reach statistical significance, as their 95% CIs all included 1. The odds ratio was 1.249 (95% CI: 0.870–1.820) for Hb, 1.231 (95% CI: 0.892–1.711) for age, 0.939 (95% CI: 0.471–1.846) for sex, and 0.912 (95% CI: 0.660–1.242) for BMI.

#### 3.3.5 Discriminative performance of NEU%, CSF-PRO, ALB, and the combined severity assessment model

**Figure 6:**
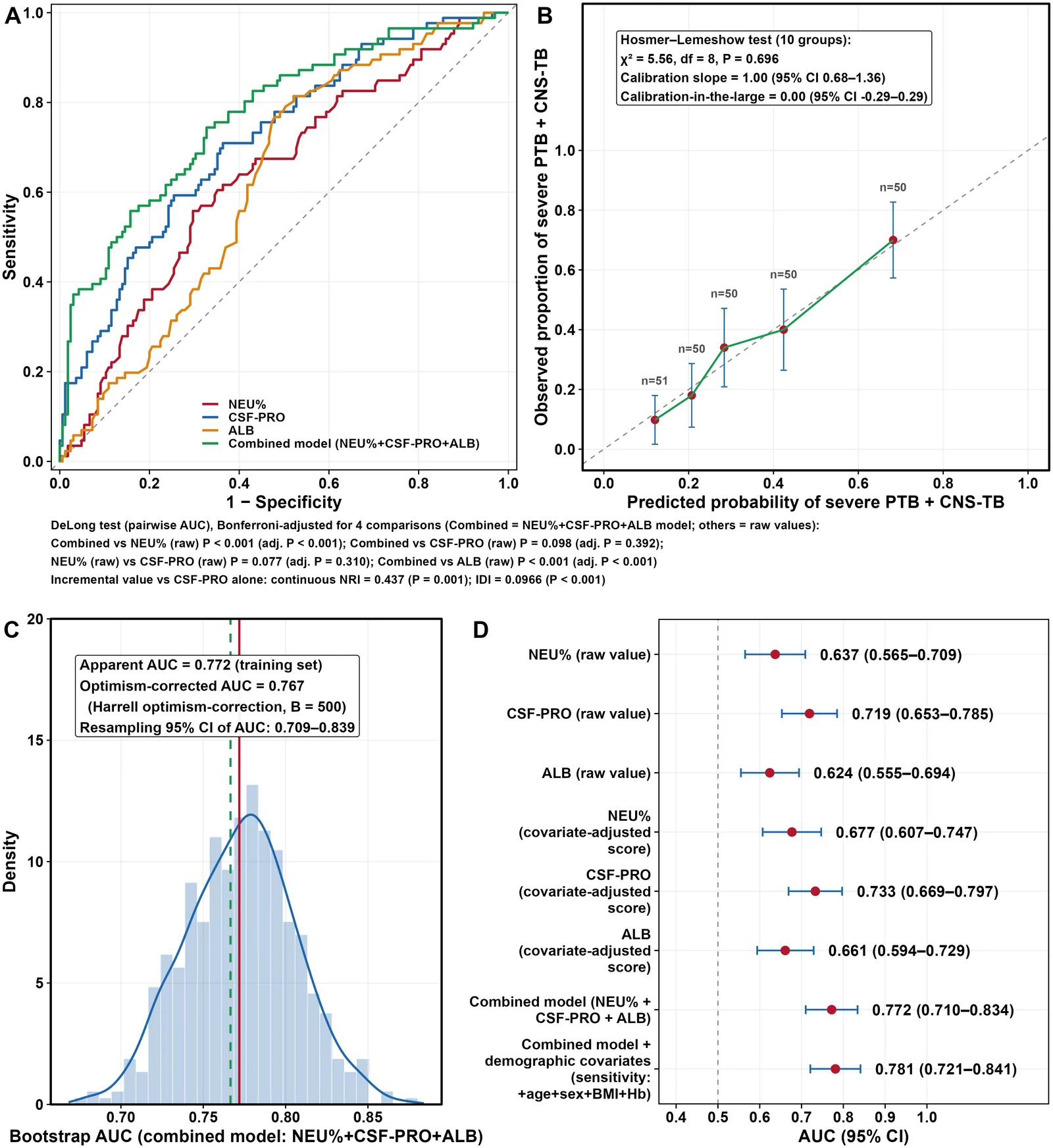
Discriminative performance and internal validation of NEU%, CSF-PRO, ALB, and the combined model for discriminating severe from mild PTB + CNS-TB. AUC, area under the receiver operating characteristic curve. **(A)** ROC curves for NEU% (raw), CSF-PRO (raw), ALB (raw), and the combined model (NEU% + CSF-PRO + ALB). The diagonal dashed line represents the line of no discrimination (AUC = 0.5). Pairwise AUC comparisons were performed using the DeLong test, with Bonferroni correction for four comparisons; unadjusted and adjusted P values are shown below the plot. **(B)** Calibration curve of the combined model, based on five predicted-probability groups. Points show the mean predicted probability versus the observed proportion of severe PTB + CNS-TB in each group, with vertical bars representing 95% confidence intervals and n denoting the number of patients per group. The diagonal dashed line represents perfect calibration. Hosmer-Lemeshow goodness-of-fit statistics, calibration slope, and calibration-in-the-large are shown in the inset. **(C)** Distribution of bootstrap-resampled AUC values for the combined model (B = 500 resamples). The red solid line indicates the apparent AUC in the training set; the green dashed line indicates the optimism-corrected AUC obtained by Harrell’s bootstrap optimism-correction method. The inset shows the apparent AUC, optimism-corrected AUC, and the 95% resampling confidence interval. **(D)** Forest plot summarizing AUC values (with 95% CI) for NEU%, CSF-PRO, and ALB in their raw form, their covariate-adjusted forms (age, sex, BMI, and Hb included as covariates), the combined model, and the combined model with added demographic covariates (sensitivity analysis). The dashed vertical line indicates AUC = 0.5.

ROC analysis was used to evaluate the discriminative performance of NEU%, CSF-PRO, ALB, and their combined severity assessment model for distinguishing severe from mild PTB + CNS-TB (**Figure 6**, **Table 4**). Among the three raw markers, CSF-PRO showed the highest AUC of 0.719 (95% CI: 0.653–0.785), with a sensitivity of 70.90% and specificity of 63.60% at the optimal cutoff of 977.05 mg/L. NEU% showed an AUC of 0.637 (95% CI: 0.565–0.709), with a sensitivity of 55.80% and specificity of 70.30% at the cutoff of 0.82. ALB showed the lowest AUC among the three markers, at 0.624 (95% CI: 0.555–0.694), with a sensitivity of 81.40% and specificity of 47.90% at the cutoff of 34.25 g/L.

**Table 4:** Discriminative performance of NEU%, CSF-PRO, ALB, the combined model, and a sensitivity model for discriminating severe from mild PTB + CNS-TB.

Marker	AUC (95% CI)	Optimal cutoff	Sensitivity (%)	Specificity (%)	PPV (%)	NPV (%)
NEU% (raw)	0.637(0.565–0.709)	0.82	55.80	70.30	49.50	75.30
CSF-PRO (raw)	0.719 (0.653–0.785)	977.05 mg/L	70.90	63.60	50.40	80.80
ALB (raw)	0.624 (0.555–0.694)	34.25 g/L	81.40	47.90	44.90	83.20
NEU% (covariate-adjusted score)	0.677 (0.607–0.747)	–	65.10	62.40	47.50	77.40
CSF-PRO (covariate-adjusted score)	0.733 (0.669–0.797)	–	58.10	77.60	57.50	78
ALB (covariate-adjusted score)	0.661 (0.594–0.729)	–	80.20	49.70	45.40	82.80
Combined model (NEU% + CSF-PRO + ALB, primary)	0.772 (0.710–0.834)	–	74.40	67.30	54.20	83.50
Sensitivity model (+ Age + Sex + BMI + Hb)	0.781 (0.721–0.841)	–	70.90	73.90	58.7	83.00

PPV, positive predictive value; NPV, negative predictive value. Covariate-adjusted scores refer to predicted probabilities from a logistic regression model containing the single marker together with age, sex, BMI, and Hb. The combined model includes NEU%, CSF-PRO, and ALB as joint predictors in a single logistic regression model and is presented as the primary result. The sensitivity model additionally includes age, sex, BMI, and Hb; the DeLong test showed no significant difference in AUC between the combined model and the sensitivity model. Optimal cutoffs were determined using the Youden index and are not applicable (—) for models reported on the predicted-probability scale.

Covariate adjustment led to modest changes in individual marker performance. The covariate-adjusted score for CSF-PRO reached an AUC of 0.733 (95% CI: 0.669–0.797), slightly higher than the raw value. The covariate-adjusted score for NEU% reached an AUC of 0.677 (95% CI: 0.607–0.747), also higher than its raw AUC. The covariate-adjusted score for ALB reached an AUC of 0.661 (95% CI: 0.594–0.729), again slightly higher than the raw ALB value. These changes suggest that part of the discriminative ability of each marker is related to baseline demographic and nutritional factors, although each marker retained discriminative ability after covariate adjustment.

The combined model integrating NEU%, CSF-PRO, and ALB achieved an AUC of 0.772 (95% CI: 0.710–0.834), with a sensitivity of 74.40% and specificity of 67.30%, numerically higher than any single raw marker (**Figure 6D**). Pairwise DeLong tests with Bonferroni adjustment for four comparisons showed that the combined model performed significantly better than NEU% alone (*P* < 0.001, adjusted *P* < 0.001) and than ALB alone (*P* < 0.001, adjusted *P* < 0.001). In contrast, the improvement of the combined model over CSF-PRO alone did not remain statistically significant after adjustment (*P* = 0.098, adjusted *P* = 0.392), and the difference between NEU% and CSF-PRO alone was also non-significant (*P* = 0.077, adjusted *P* = 0.310) (**Figure 6A**). These findings indicate that the incremental discriminative benefit of the combined model is derived mainly from its superiority over NEU% and ALB individually, rather than from a statistically confirmed advantage over CSF-PRO, which was the strongest single marker.

**Figure 7:**
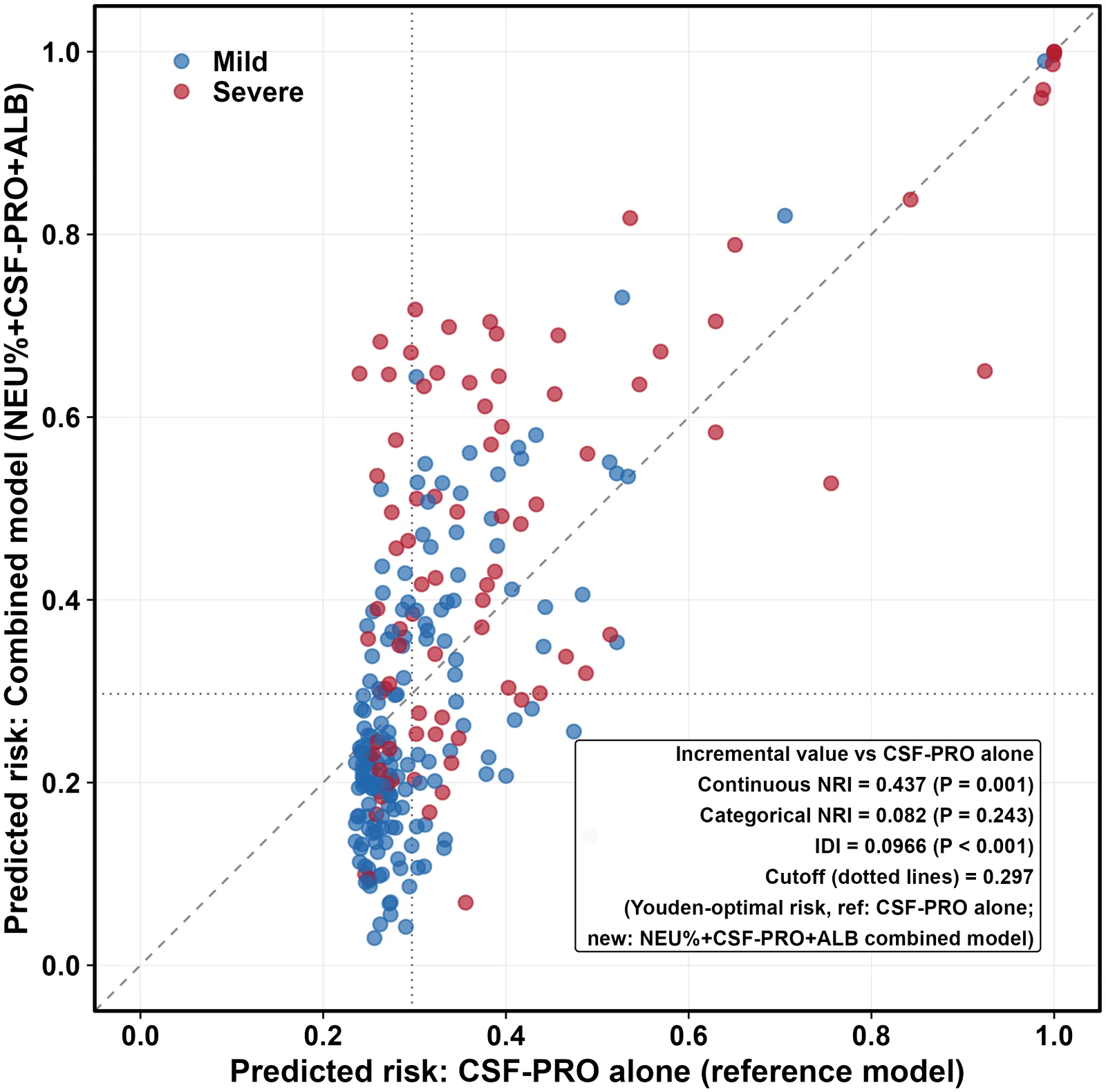
Risk reclassification plot comparing the combined model with the CSF-PRO-alone reference model. Each point represents an individual patient, with predicted risk of severe PTB + CNS-TB derived from the CSF-PRO-alone model (x-axis) plotted against predicted risk from the combined model incorporating NEU%, CSF-PRO, and ALB (y-axis). The diagonal dashed line indicates the line of identity (no change in predicted risk between models). Dotted lines denote the Youden-optimal risk cutoff used for categorical net reclassification improvement (NRI) classification. Continuous NRI, categorical NRI, and integrated discrimination improvement (IDI) were calculated to quantify the incremental predictive value of the combined model relative to the CSF-PRO-alone reference model. NRI, net reclassification improvement; IDI, integrated discrimination improvement.

To further quantify the incremental value of adding NEU% and ALB to CSF-PRO, reclassification analysis was performed using CSF-PRO alone as the reference model (**Figure 7**). The combined model yielded a significant continuous NRI of 0.437 (*P* = 0.001) and a significant IDI of 0.0966 (*P* < 0.001), indicating an overall improvement in risk discrimination and probability separation between mild and severe cases. However, the categorical NRI at the Youden-optimal cutoff of 0.297 was not statistically significant (NRI = 0.082, *P* = 0.243), suggesting that although the combined model improves continuous risk stratification, it does not substantially reclassify patients across this particular binary risk threshold. This pattern of a significant continuous NRI and IDI alongside a non-significant categorical NRI is consistent with the borderline AUC comparison against CSF-PRO alone and should be interpreted as evidence of modest rather than robust incremental value.

Calibration and internal validation of the combined model were assessed to evaluate its clinical applicability (**Figures 6B, C**). The Hosmer-Lemeshow test indicated no significant deviation between predicted and observed risk (*χ²* = 5.56, df = 8, *P* = 0.696), and the calibration slope was 1.00 (95% CI: 0.68–1.36) with a calibration-in-the-large of 0.00 (95% CI: −0.29–0.29), both close to their ideal values, supporting adequate calibration without systematic overestimation or underestimation of risk. Bootstrap internal validation with 500 resamples yielded an apparent AUC of 0.772 and an optimism-corrected AUC of 0.767 (resampling 95% CI: 0.709–0.839), indicating minimal optimism and reasonable internal robustness of the combined model.

A sensitivity model was further constructed by adding age, sex, BMI, and Hb to the combined model. This sensitivity model achieved an AUC of 0.781 (95% CI: 0.721–0.841), with a sensitivity of 70.90% and specificity of 73.90%, and a positive predictive value of 58.70% and negative predictive value of 83.00%. DeLong testing comparing the primary three marker model against this expanded seven variable model showed no significant difference in AUC (*Z* = −0.978, *P* = 0.328). This result supports the three marker combined model of NEU%, CSF-PRO, and ALB as the primary result of this study, since the additional contribution of age, sex, BMI, and Hb to discriminative performance was negligible.

## 4 Discussion

Drawing on a retrospective cohort of 4,829 hospitalized tuberculosis patients, and after eliminating demographic and nutritional confounders through PSM, the present study systematically delineates the immunoinflammatory profile that distinguishes PTB + CNS-TB patients from those with PTB alone, while further identifying independent predictors of disease severity within the PTB + CNS-TB subpopulation. Our findings suggest the following central conclusion: hematogenous dissemination of *Mycobacterium tuberculosis* from the lungs to the intracranial compartment may be associated with reduced peripheral lymphocyte/T-cell subset counts and higher neutrophil-related systemic inflammatory indices; once CNS-TB is established, however, disease severity is primarily associated with higher neutrophil-related systemic inflammatory indices and greater abnormalities in routine CSF inflammatory parameters, and peripheral T-lymphocyte subset counts fail to meaningfully discriminate between levels of clinical severity.

### 4.1 Reduced peripheral T-cell counts in PTB + CNS-TB: an immunological phenotype associated with CNS involvement

After matching, the LYMPH count was significantly lower in the PTB + CNS-TB group than in PTB controls and carried the largest standardized mean difference (SMD = 0.757) of any post-matching comparison; Consistent reductions were observed in absolute counts of CD3^+^, CD4^+^, and CD8^+^T cells, as well as in CD45^+^ lymphocytes. It is worth emphasizing that patients with HIV infection or any other established immunosuppressive condition were rigorously excluded. Consequently, the lower peripheral T-cell counts observed in this cohort were unlikely to be fully explained by the major pre-existing immunosuppressive conditions specified in the exclusion criteria, and are more likely a direct reflection of the relentless attrition that tuberculosis itself exerts on the adaptive immune system. As our group previously reported in a study of 862 patients with extrapulmonary tuberculosis, T-lymphocyte subset counts were broadly reduced in those with CNS-TB (Chen et al., 2025a). The current findings are consistent with this observation and further advance the comparative analysis by characterizing the distinct peripheral immune-inflammatory profiles of patients with PTB + CNS-TB relative to matched patients with PTB alone. Notably, the use of uncomplicated PTB patients as the comparator group, rather than other forms of extrapulmonary TB, ensures that the between-group differences more directly capture the immunological perturbations specific to the process of mycobacterial dissemination from the lung to the intracranial space, rather than the non-specific immune attrition attendant on extrapulmonary seeding per se. The seminal review by Sia and Rengarajan detailed the multiple mechanisms by which M. tuberculosis undermines T-cell responses, including suppression of antigen presentation, inhibition of neutrophil apoptosis, and expansion of regulatory T cells (Sia and Rengarajan, 2019), while functional exhaustion and impaired responsiveness of T-cell subsets further diminish immunosurveillance against hematogenous mycobacterial spread (Vats et al., 2024).Multiple lines of evidence have linked declining CD4^+^ T cell counts to a greater propensity for extrapulmonary dissemination (Jones et al., 1993; Gupta et al., 2013), and a recent study by Jiang et al. identified depletion of Th1, Tc1, and Tc17 subsets together with impaired interferon-γ production in patients with disseminated tuberculosis (Jiang et al., 2024). Considered collectively, reduced peripheral adaptive immune cell counts may be associated with an increased risk of extrapulmonary dissemination.

### 4.2 Elevated neutrophil-related inflammatory indices accompanying lymphopenia in PTB + CNS-TB

In addition to lower lymphocyte counts, PTB + CNS-TB patients had higher NLR, PLR, SII, and NEU% than matched PTB controls. NEU% showed the largest standardized mean difference among the post-matching inflammatory indices. This bidirectional shift, characterized by lymphopenia alongside neutrophilia, constitutes a distinctive immunoinflammatory signature of PTB + CNS-TB, possibly indicative of compensatory overactivation of innate immune responses in the context of reduced adaptive immune cell counts. This pattern is in close agreement with the existing literature: NLR is among the most extensively investigated composite inflammatory markers, serving not only as a diagnostic reference in tuberculosis (Cursi et al., 2023) but also correlating with disease severity and prognosis (Abakay et al., 2015; Gu et al., 2023; Chen et al., 2025a), as well as with specific patterns of radiological enhancement in spinal tuberculosis (Shan et al., 2025). Particularly noteworthy is the observation that the NLR profile in CNS-TB differs from that seen in lymph node tuberculosis (Chen et al., 2025a; Chen et al., 2025b), reinforcing the concept of immunological heterogeneity in systemic inflammatory responses across distinct anatomical sites of disease. LMR, SII, and PLR have similarly demonstrated value in tuberculosis diagnosis, assessment of inflammatory burden, prediction of mycobacterial load, and prognostic evaluation (Chen et al., 2016; Adane et al., 2022; Yu et al., 2024; Liang et al., 2026), collectively attesting to the broad applicability of composite inflammatory indices in characterizing the systemic inflammatory state of CNS-TB.

### 4.3 Neutrophilic and intrathecal inflammatory indices associated with disease severity

Within the PTB + CNS-TB cohort, a particularly striking finding emerged from the severity comparison: none of the peripheral T-lymphocyte subset parameters differed significantly between severity subgroups, either in univariable analysis or after multivariable regression with FDR correction. This indicates that, although peripheral T-cell subset counts were lower in PTB + CNS-TB than in PTB, these parameters did not further stratify clinical severity once CNS involvement was present. A plausible explanation is that peripheral T-cell subset counts may already be globally reduced in PTB + CNS-TB patients; therefore, the acute neurological injury captured by MRC grading may be driven primarily by local intracranial inflammation rather than by fluctuations in the circulating T-cell pool.

In sharp contrast to this absence of discriminatory signal from T-cell parameters, neutrophil-related indices and intrathecal inflammatory markers demonstrated robust discriminatory performance across severity strata. Multivariable regression identified NEU% (Std.*β* = 0.609), NLR (Std.*β* = 0.525), and absolute neutrophil count (Std.*β* = 0.499) as the three peripheral blood indicators with the highest standardized regression coefficients, each significantly and positively associated with disease severity; WBC (Std.*β* = 0.398) also reached statistical significance (FDR-adjusted *P* = 0.010). These findings resonate with those of Gu et al., who similarly reported that NLR was independently associated with short-term adverse outcomes in HIV-negative tuberculous meningitis patients (Gu et al., 2023).

From a pathomechanistic standpoint, the blood-brain barrier under physiological conditions rigorously excludes neutrophils and other aggressive effector cells from the central nervous system. In tuberculous meningitis, however, neutrophils breach this barrier through chemotaxis and transmigration, subsequently playing a central pathological role in immune dysregulation and tissue injury through phagocytosis, protease and reactive oxygen species release, NETosis formation, and induction of pro-inflammatory cytokine secretion; they also emerge as key biomarkers for mediating immune reconstitution inflammatory syndrome, monitoring relapse, predicting severe phenotypes such as cerebral infarction, and supporting differential diagnosis (Barnacle et al., 2024; Ma et al., 2024). Furthermore, *M. tuberculosis* reaches the central nervous system predominantly via hematogenous and lymphatic routes; upon rupture of leptomeningeal or ependymal foci with release of bacilli, a complex inflammatory network is triggered in which astrocytes, endothelial cells, and resident microglia secrete a broad array of cytokines and chemokines—centered on TNF and encompassing IL-6, IFN-γ, and CXCL10 (Kruthika, 2022). The persistently elevated peripheral neutrophil counts and NLR observed in severe patients may thus reflect both enhanced bone marrow granulopoiesis driven by infectious stimulation and a reciprocal remodeling of the peripheral immune landscape by intracranial inflammatory signals.

### 4.4 Association of routine CSF parameters with disease severity

The performance of CSF parameters in severity stratification further supports the observation that inflammatory indices are closely associated with CNS-TB disease severity. CSF protein concentration was markedly elevated in the severe subgroup (1501.60 vs. 716.40 mg/L, *P* < 0.001, SMD = 0.560) and showed the strongest standardized association among all parameters examined (Std.*β* = 0.622), suggesting greater intrathecal inflammatory burden and/or altered barrier permeability in severe disease. Shukla et al. demonstrated that increased barrier permeability in tuberculous meningitis correlates closely with the concentrations of pro-inflammatory mediators such as TNF-α, suggesting that elevated CSF protein does not simply reflect passive plasma leakage but rather represents the structural consequence of locally driven inflammatory barrier breakdown (Shukla et al., 2022). Concurrently, CSF chloride was significantly lower in the severe subgroup (117.05 vs. 120.80 mmol/L, *P* < 0.001) and showed an significant negative association with severity (Std.*β* = −0.435). This classic inverse relationship between rising CSF protein and falling CSF chloride exhibited a clear gradient separation across MRC grades in the present study, providing objective laboratory-level validation of the MRC grading system and confirming that the severity construct it defines corresponds to quantifiable, graded changes in intrathecal biochemistry.

Total CSF leukocyte count, absolute mononuclear cell count, and absolute polynuclear cell count were all significantly elevated in the severe subgroup, yet neither the proportion of mononuclear cells nor that of polynuclear cells differed between the two groups. This pattern implies that the accumulation of leukocytes in the CSF of severe patients is proportional across cell types, with mononuclear and polynuclear cells rising in tandem rather than one subset selectively expanding. Such a profile is more consistent with a generalized, non-selective influx of peripheral blood cells into the intrathecal compartment secondary to globally increased barrier permeability, rather than the targeted recruitment of a specific cell population orchestrated by discrete chemotactic signals. A recent study published in *Annals of Indian Academy of Neurology* similarly found no correlation between the CSF NLR and tuberculous meningitis severity (Mateen et al., 2025), lending independent support to the observation of proportional CSF cellular stability reported here. The elevated absolute CSF cell counts in the severe subgroup may reflect differences in overall inflammatory burden and/or barrier permeability, rather than a clear qualitative shift in intrathecal cellular composition.

### 4.5 Independently associated factors and severity assessment model

CSF protein and neutrophil percentage are compatible with altered barrier permeability and systemic inflammation, whereas serum albumin presents a more puzzling picture, its elevation in severe cases running counter to the conventional assumption that hypoalbuminemia signals poorer nutritional reserve and thus worse outcome. Several explanations may reconcile this discrepancy. Patients with more severe TBM often present with raised intracranial pressure, which can provoke vomiting and high fever, leading to dehydration and hemoconcentration; this is indirectly supported by the higher hemoglobin levels observed in the severe group, suggesting that part of the albumin elevation may reflect reduced plasma volume rather than true nutritional or hepatic synthetic status. Alternatively, patients with rapidly progressing disease may present earlier in the clinical course, before chronic consumptive hypoproteinemia develops, which could similarly mask the expected inverse relationship. These explanations remain speculative, and the mechanism underlying the albumin-severity association warrants further study.

Although lumbar puncture is invasive, albumin and neutrophil percentage are readily available blood-based markers and may provide complementary information to CSF protein. Neither marker alone showed satisfactory discrimination. The combined model incorporating NEU%, CSF protein, and albumin showed significantly better discrimination than NEU% or albumin alone (both Bonferroni-adjusted *P* < 0.001). However, its improvement over CSF protein alone was not statistically significant after Bonferroni correction (adjusted *P* = 0.392). Although continuous NRI (0.437, *P* = 0.001) and IDI (0.0966, *P* < 0.001) were significant, categorical NRI was not (0.082, *P* = 0.243). Thus, the model may offer modest additional value for continuous risk estimation beyond CSF protein, but its incremental clinical utility remains uncertain. The model showed adequate calibration and internal robustness; nevertheless, external validation in independent cohorts is needed before clinical implementation.

### 4.6 An integrated trajectory of immunopathological progression

Taken together, the findings of the present study allow us to advance a hypothesis-generating immunopathological framework for PTB + CNS-TB. Peripheral adaptive immune suppression may be associated with an immunological milieu that is permissive for mycobacterial dissemination. The elevation of serum albumin observed in more severe cases may reflect a downstream systemic consequence of this broader process, namely intracranial hypertension–induced fluid loss and hemoconcentration, rather than an independent pathogenic pathway, and its inclusion in the severity model should therefore be understood as a marker of disease intensity rather than a distinct mechanistic driver. Superimposed on this foundation, excessive innate immune activation, spearheaded by neutrophils, sustains a state of persistent systemic inflammation that may contribute to structural disruption of the blood–brain and blood–CSF barriers. The resulting increase in barrier permeability may be consistent with a two-pronged consequence: on one hand, peripheral inflammatory cells and mediators may enter the intrathecal compartment more readily; on the other, CSF protein may accumulate and chloride transport may become impaired, collectively potentially establishing a positive feedback loop of intracranial inflammation. Within this proposed cascade, disease severity may be more closely associated with the magnitude of inflammatory pathway activation than with the abundance or scarcity of circulating T cells. Existing evidence indicates that the high mortality and neurological sequelae associated with CNS-TB are both closely tied to uncontrolled progression of intracranial inflammation (Rohlwink et al., 2017; Donovan et al., 2026), which is consistent with an important role of inflammatory mechanisms in shaping clinical outcomes.

### 4.7 Limitations

The present study carries several limitations that warrant acknowledgment and should be addressed in future work. First, the retrospective, single-center design constrains the strength of causal inference and limits the generalizability of the findings to broader populations. Second, only 105 (41.83%) of the 251 PTB + CNS-TB cases were microbiologically confirmed, whereas the remaining cases were diagnosed according to clinical-radiological criteria. As no sensitivity analysis restricted to microbiologically confirmed CNS-TB cases was performed, diagnostic misclassification among clinically diagnosed cases may have influenced the estimated associations and limits the certainty and generalizability of our findings. Third, the absence of transcriptomic profiling precludes any mechanistic elucidation, at the gene expression level, of neutrophil activation, T-cell functional exhaustion, or the molecular regulation of barrier integrity. Fourth, CSF cytokine profiles (including TNF-α, IFN-γ, IL-6, and related mediators) were not routinely measured, which restricts the depth of insight into the intrathecal inflammatory cascade. Fifth, T-lymphocyte subsets were characterized solely by counts and percentages; without assessment of functional markers such as programmed death-1 expression or cytokine secretion capacity, the possibility that T-cell functional status retains latent value for severity stratification cannot be entirely excluded. Sixth, as an inherent consequence of nearest-neighbor PSM, the 954 matched PTB controls were selected from a pool of 4,578 PTB inpatients on the basis of age, sex, BMI, albumin, and hemoglobin. Compared with the 3,624 unmatched PTB patients, the matched controls showed lower BMI, albumin, hemoglobin, and CD4^+^/CD8^+^ T-cell counts, together with higher neutrophil counts and NLR (**Supplementary Table 5**). This pattern indicates that the matching algorithm preferentially retained PTB patients whose nutritional, inflammatory, and T-cell profiles were already shifted toward those of the PTB + CNS-TB group, rather than representing the broader PTB inpatient population. Consequently, the generalizability of the matched comparisons to all hospitalized PTB patients is limited. Because the matched controls were systematically more compromised across nutritional, inflammatory, and T-cell dimensions than the overall PTB cohort, the case-control contrast captured in our primary analysis is narrower than it would be against the full PTB population. This suggests that the true population-level differences in immunoinflammatory profiles between PTB + CNS-TB and PTB patients are likely larger than reported here, so this limitation, if anything, reinforces rather than undermines the robustness of our conclusions.

Seventh although PSM balanced sex, age, BMI, ALB, and Hb, residual confounding cannot be excluded. Other clinically relevant factors, including diabetes mellitus, smoking and alcohol use, previous anti-tuberculosis treatment, drug resistance, symptom duration, hepatic or renal comorbidities, and other inflammatory conditions, were not included in the primary matching model because their exposure levels and severity could not be uniformly characterized in this retrospective cohort. Crude categorization may have introduced misclassification and unstable estimates in small subgroups. Patients with HIV infection, autoimmune disease, malignancy, or recent immunosuppressive therapy were excluded *a priori*, thereby minimizing major sources of immunological confounding; however, confounding from milder or unmeasured comorbidities may remain. In addition, ALB and Hb may reflect not only pre-admission nutritional or chronic disease status but also disease-related processes. Therefore, the achieved covariate balance and observed associations should be interpreted cautiously. Future studies incorporating standardized comorbidity assessment and longitudinal pre-admission measurements are warranted.

Notwithstanding these limitations, the study carries meaningful implications for clinical translation. In patients with PTB + CNS-TB, peripheral blood NLR and NEU offer a practical, low-cost first-line tool for gauging disease severity, one that is particularly well-suited to resource-limited settings. Combined monitoring of CSF protein and chloride, in turn, facilitates objective corroboration and longitudinal tracking of MRC grade. Conversely, while peripheral T-lymphocyte subset counts provide a useful window into the patient’s overall immune background, they should not be used as direct surrogates for CNS-TB severity stratification. Future research should integrate transcriptomic analyses and CSF cytokine data within prospective, multicenter cohorts to rigorously validate the proposed pathological sequence, in which adaptive immune suppression and innate immune hyperactivation lead to barrier disruption, which in turn amplifies intracranial inflammation, thereby building a more robust evidence base for precision risk stratification and targeted therapeutic intervention in CNS-TB.

## 5 Conclusion

PTB + CNS-TB was associated with a distinctive peripheral immunoinflammatory profile characterized by lower T-lymphocyte counts and higher neutrophil-related inflammatory indices. Among patients with PTB + CNS-TB, disease severity was more closely associated with neutrophil-related systemic inflammatory indices and routine CSF parameters than with peripheral T-lymphocyte subset counts. NEU%, CSF protein, and serum albumin were independently associated with severe disease in the multivariable analysis. The internally validated model incorporating these variables showed moderate discriminatory performance and requires external validation before clinical implementation.

## Data Availability

The datasets used and/or analyzed during the current study are available from the first author upon reasonable request. Requests to access these datasets should be directed to 497795218@qq.com.

## Glossary

ALB: albumin
ATT: average treatment effect on the treated
BMI: body mass index
CI: confidence interval
CNS-TB: central nervous system tuberculosis
CSF: cerebrospinal fluid
CSF-Cl: cerebrospinal fluid chloride
CSF-GLU: cerebrospinal fluid glucose
CSF-MNC: cerebrospinal fluid mononuclear cell count
CSF-MNC%: cerebrospinal fluid mononuclear cell percentage
CSF-PMN: cerebrospinal fluid polymorphonuclear cell count
CSF-PMN%: cerebrospinal fluid polymorphonuclear cell percentage
CSF-PRO: cerebrospinal fluid protein
CSF-WBC: cerebrospinal fluid white blood cell count
ESS: effective sample size
FDR: false discovery rate
Hb: hemoglobin
HIV: human immunodeficiency virus
IPTW: inverse probability of treatment weighting
IQR: interquartile range
LMR: lymphocyte-to-monocyte ratio
LYMPH: lymphocyte
*M*. tuberculosis: *Mycobacterium tuberculosis*
MONO: monocyte
MRC: Medical Research Council
NEU: neutrophil count
NEU%: neutrophil percentage
NLR: neutrophil-to-lymphocyte ratio
PLR: platelet-to-lymphocyte ratio
PLT: platelet
PSM: propensity score matching
PTB: pulmonary tuberculosis
PTB + CNS-TB: pulmonary tuberculosis complicated by central nervous system tuberculosis
RBC: red blood cell
SD: standard deviation
SII: systemic immune-inflammation index
SMD: standardized mean difference
Std.β: standardized regression coefficient
TB: tuberculosis
WBC: white blood cell count.
