# Supplementary material for: Immune-inflammatory profiles and disease severity in pulmonary tuberculosis complicated by central nervous system tuberculosis
Source: Front Cell Infect Microbiol. 2026 Sep 9;16:1899254. doi: 10.3389/fcimb.2026.1899254 (PMC13597226; doi:10.3389/fcimb.2026.1899254)
Supplement: Supplementary file 1 [file Table1.docx]

Supplementary Material

**Figure S1. Propensity Score Distributions Before and After 1:4 Nearest-Neighbor Matching.**

**Note:** Density plots show the distributions of propensity scores for patients with PTB + CNS-TB and PTB before and after matching. The post-matching density curves incorporate matching weights generated by MatchIt.

**Figure S2. Covariate Balance Before and After Propensity Score Matching.**

**Note:** Love plot showing absolute standardized mean differences for age, sex, BMI, albumin, and hemoglobin before and after propensity score matching. The vertical dashed line indicates the prespecified threshold of 0.1. All matching covariates had absolute SMDs < 0.1 after matching, indicating adequate balance in the measured matching covariates.

**Figure S3. Spearman correlation matrix of peripheral blood and cerebrospinal fluid laboratory parameters in patients with PTB + CNS-TB.**

**Note:** Cell values indicate Spearman's ρ, with color intensity representing the strength and direction of correlation (red, positive; blue, negative). Asterisks denote two-sided significance levels (*P < 0.05, **P < 0.01, ***P < 0.001).

 **Figure S4. Hierarchical clustering of peripheral blood and cerebrospinal fluid laboratory parameters based on Spearman correlation in patients with PTB + CNS-TB.**

**Note:** Clustering was performed based on a distance metric of 1 minus the absolute Spearman correlation coefficient. Branch height reflects the dissimilarity between variables; variables joined at low height are highly correlated.

**Figure S5. Ten-fold cross-validation curve for LASSO logistic regression in selection of candidate predictors of severe PTB + CNS-TB.**

**Note:** Each point represents the mean binomial deviance at a given λ, with vertical bars indicating the cross-validation error range. The left dashed line marks λ_min (minimum mean deviance); the right dashed line marks λ_1SE (largest λ within one standard error of the minimum).

**Table S1. Clinical Parameters of Patients With PTB + CNS-TB and PTB Before Propensity Score Matching**

| **Variable** | **PTB+CNS-TB (n = 251)** | **PTB (n = 4578)** | **SMD** | **Statistic** | ***P* value** |
| --- | --- | --- | --- | --- | --- |
| Sex (Male), n (%) | 169 (67.3%) | 3292 (71.9%) | 0.100 | *χ²* = 2.457 | 0.117 |
| Age,M(Q₁,Q₃) | 56.00 (36.00,67.00) | 57.00 (42.00,68.00) | 0.103 | *W* = 540634.500 | 0.115 |
| BMI ,M(Q₁,Q₃) | 19.22 (17.83,21.33) | 20.38 (18.42,22.58) | 0.330 | *W* = 467090.000 | < 0.001 |
| ALB,M(Q₁,Q₃) | 35.90 (32.25,39.57) | 39.70 (36.80,42.30) | 0.662 | *W* = 357408.000 | < 0.001 |
| Hb,M(Q₁,Q₃) | 115.00 (100.00,126.00) | 128.00 (116.00,139.00) | 0.662 | *W* = 357689.000 | < 0.001 |
| WBC,M(Q₁,Q₃) | 6.81 (5.12,8.85) | 6.55 (5.33,8.11) | 0.112 | *W* = 603335.500 | 0.181 |
| NEU,M(Q₁,Q₃) | 5.33 (3.60–7.31) | 4.16 (3.14,5.70) | 0.364 | *W* = 706141.500 | < 0.001 |
| NEU%,M(Q₁,Q₃) | 0.79 (0.69,0.85) | 0.65 (0.57,0.74) | 0.997 | *W* = 875338.500 | < 0.001 |
| LYMPH,M(Q₁,Q₃) | 0.81 (0.54,1.22) | 1.50 (1.13,1.95) | 1.041 | *W* = 241946.000 | < 0.001 |
| MONO,M(Q₁,Q₃) | 0.46 (0.32,0.65) | 0.46 (0.36,0.61) | 0.021 | *W* = 565071.000 | 0.660 |
| RBC ,M(Q₁,Q₃) | 4.02 (3.59,4.43) | 4.34 (3.98,4.72) | 0.072 | *W* = 415517.500 | < 0.001 |
| PLT,M(Q₁,Q₃) | 249.00 (195.00,319.50) | 255.00 (207.00,313.00) | 0.048 | *W* = 555074.000 | 0.365 |
| NLR,M(Q₁,Q₃) | 6.42 (3.49,11.12) | 2.71 (1.80,4.53) | 0.358 | *W* = 884306.000 | < 0.001 |
| PLR,M(Q₁,Q₃) | 302.33 (195.85,479.98) | 168.69 (120.92,245.50) | 0.502 | *W* = 866169.000 | < 0.001 |
| LMR,M(Q₁,Q₃) | 1.78 (1.13–3.04) | 3.27 (2.12,4.68) | 0.587 | *W* = 306622.000 | < 0.001 |
| SII,M(Q₁,Q₃) | 1621.93 (853.00,2792.18) | 679.10 (410.24,1302.75) | 0.295 | *W* = 837016.000 | < 0.001 |
| CD3^+^%,M(Q₁,Q₃) | 70.00 (61.00,76.00) | 71.00 (64.00,76.00) | 0.147 | *W* = 532881.500 | 0.053 |
| CD3^+^,M(Q₁,Q₃) | 697.00 (373.50,979.00) | 1131.00 (822.00,1492.00) | 0.841 | *W* = 284422.500 | < 0.001 |
| CD4^+^%,M(Q₁,Q₃) | 38.00 (30.00,45.00) | 41.00 (35.00,47.00) | 0.344 | *W* = 468765.500 | < 0.001 |
| CD4^+^,M(Q₁,Q₃) | 357.00 (208.50,573.50) | 659.00 (467.00,885.00) | 0.898 | *W* = 277362.000 | < 0.001 |
| CD8^+^%,M(Q₁,Q₃) | 27.00 (20.00,35.00) | 25.00 (20.00,32.00) | 0.174 | *W* = 620022.500 | 0.034 |
| CD8^+^,M(Q₁,Q₃) | 244.00 (141.00,400.50) | 403.00 (277.00,568.00) | 0.525 | *W* = 344620.500 | < 0.001 |
| CD4^+^/CD8^+^,M(Q₁,Q₃) | 1.39 (0.96,2.12) | 1.61 (1.19,2.18) | 0.032 | *W* = 493733.000 | < 0.001 |
| CD45^+^,M(Q₁,Q₃) | 1030.00 (563.00,1392.50) | 1630.50 (1223.00,2105.00) | 0.848 | *W* = 280468.000 | < 0.001 |

**Note:**Data are presented as median (IQR) for non-normally distributed variables and n (%) for categorical variables. Before matching, P values were calculated using Wilcoxon rank-sum test and chi-square test. SMD, standardized mean difference. For this unmatched comparison, absolute standardized mean differences (SMDs) were calculated using the pooled standard deviation of the PTB+CNS-TB and PTB groups as the standardizing denominator. PTB, pulmonary tuberculosis; CNS-TB, central nervous system tuberculosis; PSM, propensity score matching; BMI, body mass index; ALB, albumin (g/L); Hb, hemoglobin (g/L); WBC, white blood cell (×10⁹/L); NEU, neutrophil (×10⁹/L); NEU%, neutrophil percentage; LYMPH, lymphocyte (×10⁹/L); MONO, monocyte (×10⁹/L); RBC, red blood cell (×10^12^/L); PLT, platelet (×10⁹/L); NLR, neutrophil-to-lymphocyte ratio; PLR, platelet-to-lymphocyte ratio; LMR, lymphocyte-to-monocyte ratio; SII, systemic immune-inflammation index (calculated from neutrophil, platelet, and lymphocyte counts). Lymphocyte subset analysis: CD3^+^ indicates total T lymphocytes (cells/μL, %); CD3^+^% indicates the percentage of CD3^+^ T lymphocytes relative to total lymphocyte population; CD4^+^ indicates helper T cells (cells/μL, %); CD4^+^% indicates the percentage of CD4^+^ T lymphocytes relative to total lymphocyte population; CD8^+^ indicates cytotoxic T lymphocytes (cells/μL, %); CD8^+^% indicates the percentage of CD8^+^ T lymphocytes relative to total lymphocyte population; CD4^+^/CD8^+^ indicates the ratio of CD4^+^ to CD8^+^ T lymphocytes; CD45^+^ indicates total lymphocytes (cells/μL).

**Table S2. Covariate balance before and after inverse probability weighting**

| **Variable** | **SMD unweighted** | **SMD weighted** |
| --- | --- | --- |
| Age | 0.098 | 0.017 |
| Sex | 0.098 | 0.012 |
| BMI | 0.351 | 0.008 |
| ALB | 0.607 | 0.011 |
| Hb | 0.629 | 0.009 |

**Note:** Balance was assessed for the covariates included in the propensity-score model: age, sex, body mass index, albumin, and hemoglobin. In the inverse probability of treatment weighting (IPTW) sensitivity analysis, propensity-score weights were estimated for the average treatment effect in the treated (ATT), with PTB+CNS-TB defined as the treated group. Both unweighted and weighted absolute standardized mean differences (SMDs) were calculated using the standard deviation of the PTB+CNS-TB group as the standardizing denominator, consistent with the ATT estimand. Therefore, the unweighted SMDs in this table are not expected to be identical to those in Supplementary Table S1, in which pooled standard deviations were used. Weights were truncated at the 1st and 99th percentiles. An absolute SMD <0.10 indicated adequate covariate balance. After weighting, all five covariates had absolute SMDs <0.10.

**Table S3. Weight distribution and effective sample size in the inverse probability weighting sensitivity analysis**

| **Group** | **Actual_N** | **Sum_weights** | **ESS** | **ESS_percent** | **Mean_weight** | **SD_weight** | **Min_weight** | **Max_weight** |
| --- | --- | --- | --- | --- | --- | --- | --- | --- |
| PTB + CNS-TB | 251 | 251.00 | 251 | 100.0% | 1.000 | 0.000 | 1.000 | 1 |
| PTB | 4,578 | 251.28 | 2,027 | 44.3% | 0.055 | 0.062 | 0.009 | 1 |

**Note:** Weights were estimated from the propensity score for membership in the PTB+CNS-TB group, conditional on age, sex, body mass index, albumin, and hemoglobin. The PTB group was reweighted to approximate the covariate distribution of the PTB+CNS-TB group. Weights were truncated at the 1st and 99th percentiles. The effective sample size (ESS) was calculated as $\left( \sum w)^{2}/ \sum w^{2} \right.$. ESS reflects the precision retained after weighting and does not represent the number of enrolled participants. ESS, effective sample size.

**Table S4. Inverse probability weighting sensitivity analysis of clinical and immunological parameters in patients with PTB+CNS-TB and PTB**

| **Variable** | **PTB + CNS-TB** | **PTB** | **SMD** | **Statistic** | **P value** | **Q value (FDR)** |
| --- | --- | --- | --- | --- | --- | --- |
| Sex (Male), n (%) | 67.3% | 67.9% | 0.012 | *t* = -0.183 | 0.855 | 0.932 |
| Age,M(Q₁,Q₃) | 56.00 (36.00,67.00) | 56.00 (39.00,67.00) | 0.017 | *t* = -0.307 | 0.759 | 0.867 |
| BMI ,M(Q₁,Q₃) | 19.22 (17.82,21.41) | 19.36 (17.53,21.51) | 0.007 | *t* = -0.028 | 0.978 | 0.978 |
| ALB,M(Q₁,Q₃) | 35.90 (32.20,39.64) | 36.70 (32.10,40.00) | 0.011 | *t* = -0.582 | 0.561 | 0.708 |
| Hb,M(Q₁,Q₃) | 115.00 (100.00,126.00) | 115.00 (100.00–128.00) | 0.009 | *t* = -0.501 | 0.617 | 0.740 |
| WBC,M(Q₁,Q₃) | 6.81 (5.09,8.86) | 6.82 (5.46,8.83) | 0.067 | *t* = -0.743 | 0.457 | 0.610 |
| NEU,M(Q₁,Q₃) | 5.33 (3.60,7.36) | 4.64 (3.34,6.68) | 0.097 | *t* = 2.666 | 0.008 | 0.014 |
| NEU%,M(Q₁,Q₃) | 0.79 (0.68,0.85) | 0.70 (0.59,0.79) | 0.663 | *t* = 10.325 | < 0.001 | < 0.001 |
| LYMPH,M(Q₁,Q₃) | 0.81 (0.54,1.22) | 1.34 (0.94,1.81) | 0.760 | *t* = -12.326 | < 0.001 | < 0.001 |
| MONO,M(Q₁,Q₃) | 0.46 (0.32,0.65) | 0.50 (0.37,0.68) | 0.180 | *t* = -2.522 | 0.012 | 0.018 |
| RBC ,M(Q₁,Q₃) | 4.02 (3.59,4.43) | 4.06 (3.62,4.47) | 0.089 | *t* = -0.060 | 0.952 | 0.978 |
| PLT,M(Q₁,Q₃) | 249.00 (195.00,320.00) | 278.00 (220.00,362.00) | 0.300 | *t* = -4.317 | < 0.001 | < 0.001 |
| NLR,M(Q₁,Q₃) | 6.42 (3.46,11.17) | 3.51 (2.07,6.55) | 0.271 | *t* = 10.721 | < 0.001 | < 0.001 |
| PLR,M(Q₁,Q₃) | 302.33 (195.76,480.65) | 206.67 (138.95,336.62) | 0.333 | *t* = 8.814 | < 0.001 | < 0.001 |
| LMR,M(Q₁,Q₃) | 1.78 (1.13,3.05) | 2.59 (1.56,4.06) | 0.335 | *t* = -7.867 | < 0.001 | < 0.001 |
| SII,M(Q₁,Q₃) | 1621.93 (835.00,2806.93) | 961.95 (493.40,2154.77) | 0.196 | *t* = 7.477 | < 0.001 | < 0.001 |
| CD3^+^%,M(Q₁,Q₃) | 70.00 (61.00,76.00) | 71.00 (64.00,77.00) | 0.193 | *t* = -2.584 | 0.010 | 0.017 |
| CD3^+^,M(Q₁,Q₃) | 697.00 (373.00,981.00) | 1023.00 (704.00,1391.00) | 0.626 | *t* = -10.569 | < 0.001 | < 0.001 |
| CD4^+^%,M(Q₁,Q₃) | 38.00 (30.00,45.00) | 41.00 (34.00,47.00) | 0.311 | *t* = -4.231 | < 0.001 | < 0.001 |
| CD4^+^,M(Q₁,Q₃) | 357.00 (207.00,576.00) | 592.00 (391.00,823.00) | 0.673 | *t* = -11.126 | < 0.001 | < 0.001 |
| CD8^+^%,M(Q₁,Q₃) | 27.00 (20.00,35.00) | 26.00 (21.00,33.00) | 0.088 | *t* = 0.782 | 0.434 | 0.610 |
| CD8^+^,M(Q₁,Q₃) | 244.00 (141.00,402.00) | 373.00 (238.00,536.00) | 0.395 | *t* = -7.906 | < 0.001 | < 0.001 |
| CD4^+^/CD8^+^,M(Q₁,Q₃) | 1.39 (0.96,2.12) | 1.58 (1.12,2.14) | 0.029 | *t* = -2.542 | 0.011 | 0.018 |
| CD45^+^,M(Q₁,Q₃) | 1030.00 (561.00,1394.00) | 1464.00 (1031.00,1954.00) | 0.605 | *t* = -10.032 | < 0.001 | < 0.001 |

**Note:** Inverse probability weights were estimated using a propensity-score model including age, sex, body mass index, albumin, and hemoglobin. The weighting approach targeted the covariate distribution of the PTB+CNS-TB group; thus, PTB participants were reweighted to improve comparability with the PTB+CNS-TB group. Weights were truncated at the 1st and 99th percentiles. Values are presented as weighted median (interquartile range) for continuous variables and weighted percentage for sex. Continuous variables were compared using IPTW-weighted linear regression with design-based robust standard errors; variables with non-normal distributions were rank-transformed before regression. Sex was analyzed using IPTW-weighted logistic regression with robust standard errors. P values were adjusted for multiple comparisons using the Benjamini–Hochberg false discovery rate procedure. SMDs for age, sex, body mass index, albumin, and hemoglobin are balance diagnostics; SMDs for other clinical and immunological parameters describe between-group differences and should not be interpreted as measures of covariate balance. FDR, false discovery rate.

**Table S5. Clinical and Laboratory Characteristics of Matched Versus Unmatched PTB Controls**

| **Variable** | **Matched PTB (n = 954)** | **Unmatched PTB (n = 3624)** | **Statistic** | ***P* value** |
| --- | --- | --- | --- | --- |
| Sex (Male), n (%) | 644 (67.51%) | 2648 (73.07%) | *χ²* = 11.297 | < 0.001 |
| Age,M(Q₁,Q₃) | 56.00 (39.00, 68.00) | 58.00 (42.00, 68.00) | *W* = 1668329.5 | 0.097 |
| BMI,M(Q₁,Q₃) | 19.53 (17.72, 21.88) | 20.55 (18.64, 22.83) | *W* = 1420192.0 | < 0.001 |
| ALB,M(Q₁,Q₃) | 37.00 (32.83, 39.90) | 40.30 (37.60, 42.70) | *W* = 986684.5 | < 0.001 |
| Hb,M(Q₁,Q₃) | 116.00 (104.00, 129.00) | 130.00 (119.00, 140.00) | *W* = 988893.0 | < 0.001 |
| WBC,M(Q₁,Q₃) | 6.79 (5.57, 8.77) | 6.49 (5.28, 7.98) | *W* = 1916196.5 | < 0.001 |
| NEU,M(Q₁,Q₃) | 4.51 (3.41, 6.62) | 4.09 (3.09, 5.50) | *W* = 1984099.5 | < 0.001 |
| NEU%,M(Q₁,Q₃) | 0.69 (0.59, 0.78) | 0.64 (0.57, 0.73) | *W* = 2067799.5 | < 0.001 |
| LYMPH,M(Q₁,Q₃) | 1.35 (0.96, 1.83) | 1.54 (1.17, 1.97) | *W* = 1430544.5 | < 0.001 |
| MONO,M(Q₁,Q₃) | 0.51 (0.37, 0.67) | 0.45 (0.35, 0.59) | *W* = 1964270.0 | < 0.001 |
| RBC,M(Q₁,Q₃) | 4.09 (3.70, 4.50) | 4.40 (4.06, 4.76) | *W* = 1192206.0 | < 0.001 |
| PLT,M(Q₁,Q₃) | 279.50 (225.00, 359.00) | 249.00 (203.00, 303.25) | *W* = 2088042.5 | < 0.001 |
| PLR,M(Q₁,Q₃) | 208.68 (140.50, 324.43) | 159.82 (118.05, 225.45) | *W* = 2176400.5 | < 0.001 |
| NLR,M(Q₁,Q₃) | 3.46 (2.07, 6.14) | 2.60 (1.76, 4.15) | *W* = 2096702.0 | < 0.001 |
| LMR,M(Q₁,Q₃) | 2.55 (1.63, 4.03) | 3.42 (2.28, 4.82) | *W* = 1309145.0 | < 0.001 |
| SII,M(Q₁,Q₃) | 962.21 (508.91, 2062.33) | 627.12 (395.53, 1154.71) | W = 2154070.5 | < 0.001 |
| CD3^+^,M(Q₁,Q₃) | 1052.50 (738.25, 1417.75) | 1146.00 (857.00, 1504.25) | *W* = 1522890.5 | < 0.001 |
| CD3^+^%,M(Q₁,Q₃) | 72.00 (64.00, 77.00) | 71.00 (64.00, 76.00) | *W* = 1804011.5 | 0.038 |
| CD4^+^,M(Q₁,Q₃)) | 601.50 (413.00, 841.00) | 672.50 (482.00, 900.00) | *W* = 1520027.5 | < 0.001 |
| CD4^+^%,M(Q₁,Q₃) | 41.00 (34.00, 47.00) | 41.00 (35.00, 47.00) | *W* = 1721075.5 | 0.835 |
| CD8^+^,M(Q₁,Q₃) | 374.00 (247.00, 541.50) | 412.00 (284.00, 574.25) | *W* = 1572431.0 | < 0.001 |
| CD8^+^%,M(Q₁,Q₃) | 26.00 (20.00, 32.00) | 25.00 (20.00, 31.00) | *W* = 1796947.0 | 0.060 |
| CD4^+^/CD8^+^,M(Q₁,Q₃) | 1.59 (1.14, 2.16) | 1.62 (1.19, 2.19) | *W* = 1666728.0 | 0.088 |
| CD45^+^,M(Q₁,Q₃) | 1508.50 (1078.50, 1980.75) | 1656.50 (1266.75, 2132.00) | *W* = 1491141.0 | < 0.001 |
